# Impact of PepT1 deletion on microbiota composition and colitis requires multiple generations

**DOI:** 10.1038/s41522-020-0137-y

**Published:** 2020-07-21

**Authors:** Emilie Viennois, Adani Pujada, Junsik Sung, Chunhua Yang, Andrew T. Gewirtz, Benoit Chassaing, Didier Merlin

**Affiliations:** 1 Institute for Biomedical Sciences, Center for Inflammation, Immunity and Infection, Digestive Disease Research Group, Georgia State University, Atlanta, GA USA; 2 Center of Research on Inflammation, U1149 INSERM, Paris, France; 3 University of Paris, Paris, France; 4 Neuroscience Institute, Georgia State University, Atlanta, GA USA; 5 INSERM, U1016, team “Mucosal microbiota in chronic inflammatory diseases”, Paris, France; 6 Veterans Affairs Medical Center, Decatur, GA USA

**Keywords:** Microbiome, Cellular microbiology, Cellular microbiology

## Abstract

Numerous studies of knockout mice find impacts on microbiota composition that influence host phenotype. However, such differences can vanish when KO mice are compared directly to WT littermates, suggesting these differences do not reflect the genetic deletion per se but microbiota composition drifting over generations. Hence, our hypothesis that absence of di/tri-peptide transporter PepT1 altered microbiota composition resulting in resistance to colitis compelled scrutiny. In this study, we used PepT1^−/−^ and WT founder mice bred separately for multiple generations. Such mice were then bred to each other to generate F1 PepT1^−/−^ and WT littermates, which were then bred within their genotype to generate F2, F3, and F4, offspring. Here we report that founder PepT1^−/−^ mice were, relative to their WT counterparts, resistant to DSS colitis. Such resistance was associated with alterations in gut microbiota, which, when transplanted to germfree mice, was sufficient to transfer resistance to colitis. Such differences were not observed when comparing F1 PepT1^−/−^ to F1 WT littermates but rather, returned gradually over subsequent generations such that, relative to their F4 WT controls, F4 PepT1^−/−^ displayed microbiota composition and colitis-resistant phenotype nearly identical to the founder PepT1^−/−^ mice. Our findings indicate a role for PepT1 in influencing microbiota composition and, consequently, proneness to colitis and cancer. Overall, our study indicates that littermate-controlled experiments can be insufficient for assessing microbiota-dependent phenotypes and prevent a full comprehension of genotype-driven phenomena. Rather, impact of a single genetic alteration on microbiota and host phenotype may take generations to manifest.

## Introduction

Peptide transporter 1 (PepT1), encoded by the S*LC15A1* gene, is an apical proton-coupled oligopeptide transporter that mediates the uptake of a wide range of dietary and bacterial di- or tripeptide substrates into intestinal epithelial cells^1^. PepT1 is solely responsible for absorption of di/tripeptides arising from dietary proteins and also transport various peptidomimetics like β-lactam antibiotics, protease inhibitors, and antivirals^2^.

There is considerable evidence in the literature identifying PepT1 as a key contributor to the development and progression of inflammatory bowel disease (IBD)^3–5^. While PepT1 expression is normally restricted to the small intestine, our studies in mice show that overexpression of human PepT1 in the colon exacerbates experimental colitis, whereas a PepT1 deficiency (PepT1^−/−^ mice) is sufficient to protect against dextran sulfate sodium (DSS)-induced colitis and colitis-associated cancer (CAC)^5,6^. Increased PepT1 levels were observed in colon biopsies from patients IBD and colorectal carcinoma^6,7^.

IBD is multifactorial disease involving environmental and genetic factors that lead to dysfunction of the epithelial barrier with subsequent deregulation of the mucosal immune system and seemingly inappropriate immune responses against the gut microbiota^8–10^. While microbiota composition is generally viewed as an environmental factor, recent studies have demonstrated that host genetics influence the gut microbiome^11,12^. In this context, we sought to investigate the role played by the microbiota in the protective phenotype against intestinal inflammation observed in PepT1^−/−^ mice. Our experiments were initially performed using separately bred and housed wild-type (WT) and PepT1^−/−^ mice, as was done in our previous studies^5,6^. We found that fecal transplants, from PepT1^−/−^ mice but not WT control mice, conferred protection against DSS-induced colitis and CAC to WT germ-free (GF) mice. However, recent studies have demonstrated that failure to compare genetically modified mice to their WT littermates can produce misleading conclusions^13–15^. Accordingly, we used WT and PepT1^−/−^ littermates (F1) from PepT1^+/−^ crosses (F0-het) to investigate the effects of a PepT1 deficiency on the microbiota and its causal relationship to DSS susceptibility. Interestingly, F1 generation PepT1^−/−^ mice showed no greater resistance to DSS-induced colitis than their WT littermates; however, the protective effect of a PepT1 deficiency was progressively restored in the descendants of littermates (F2-F4). The gradual restoration of the protective phenotype also correlated with progressive changes in the microbiota across generations. Together, these findings demonstrate the potential of PepT1 genotype to shape microbiota and impact colitis susceptibility. More generally, our findings show that exclusive use of littermate comparison can mask genotype-driven phenotypes that take more than one generation to establish.

## Results

### Microbiota function is altered in PepT1^−/−^ mice

**Fig. 1:**
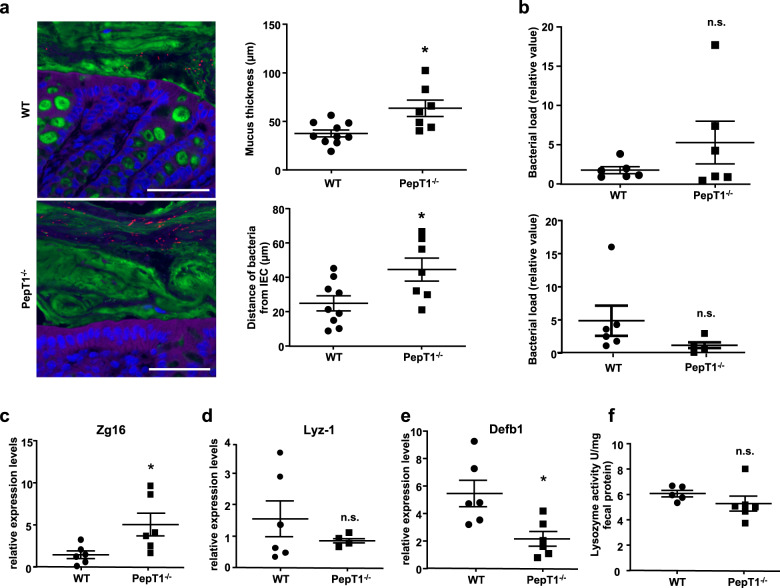
The microbiota function of PepT1^−^/^−^ mice is altered. Colon tissues and feces were collected from 6-month-old PepT1^−/−^ and WT mice. **a** Representative images of confocal microscopy analyses of microbiota localization in Carnoy-fixed colonic tissues of WT and PepT1^−/−^ mice, showing mucus thickness (upper right) and distance of the closest bacteria to the intestinal epithelium (lower right) per genotype across five high-powered fields. Green, Muc2; purple, actin; red, bacteria; blue, DNA. Scale bar: 10 μm. **b** PCR-based quantification of bacterial load in feces (upper) and adhered to the mucosa (lower). **c**–**e** Total RNA was extracted from colons, and the expression levels of Zg16 (**c**), Lyz1 (**d**) and Defb1 (**e**) were quantified by qPCR and normalized to those of 36B4. **f** Enzymatic activity of lysozyme against peptidoglycan in feces supernatants. Data are presented as the mean ± SEM. Significance was determined by unpaired two-tailed student’s *t-*test (*n* = 5–6 mice/group; *P < 0.05, n.s. non-significant).

PepT1^−/−^ are reported to be relatively resistant to experimental colitis. We hypothesize that protection might be driven by gut microbiota. Hence, we first analyzed one parameter through which the microbiota could be more or less harmful to the host, namely bacterial encroachment. We determined that mucus thickness and the average distance of bacteria from the intestinal epithelium were increased in PepT1^−/−^ mouse colons compared with their WT counterparts (Fig. 1a). Bacterial loads in colon seemed to be affected in PepT1^−/−^ as we observed tendencies to increased fecal and decreased mucus-associated bacterial loads in PepT1^−/−^ mice compared with WT mice (Fig. 1b). The host uses an arsenal of techniques to keep the microbiota at a safe distance from the epithelium and such techniques contribute to resistance against the microbiota. For example, expression of the gene encoding the lectin-like protein ZG16 (zymogen granule protein 16), an abundant mucus component that participates in maintaining bacteria at a safe distance from the intestinal epithelium^16^, was increased in PepT1^−/−^ mice compared with WT mice (Fig. 1c). However, expression levels of Muc2 (mucin 2), encoding the main mucus protein MUC2, was unchanged (data not shown). The increased mucus layer thickness observed in PepT1^−/−^ colons could also be a consequence of an increase number of goblet cells, which are the mucus-producing cells^17^. Indeed, staining with periodic acid and Alcian blue revealed an increased number of goblet cells per crypt in PepT1^−/−^ colons compared with those of WT mice, as well as an altered distribution of these cells (Supplementary Fig. 1a and b). A defect in Paneth cells, which produce anti-microbial peptides such as α-defensin and lysozyme that impact bacteria and protect the intestinal mucosa^18,19^, can alter the microbiota and affect homeostasis^20^. Hence, we investigated Paneth cell content and activity using the marker lysozyme-1. We found no change in either the expression (Fig. 1d) or activity (Fig. 1f) of this marker. The colonic epithelium additionally expresses constitutively produced β-defensins that have anti-microbial activity. We analyzed the expression of defensin β1, which can be retained in the mucus to help keep the microbiota at a safe distance from the epithelium^21^, and found that its expression level was lower in PepT1^−/−^ mice compared to WT mice (Fig. 1e). This could reflect a lower activation of epithelial defensin production due to the microbiota being more distant from the mucosal layer in PepT1^−/−^ mice than in WT mice (Fig. 1a). Another measure of microbiota healthiness is an assessment of its pro-inflammatory potential using fecal lipopolysaccharide (LPS) and flagellin (FliC) that we found unchanged in PepT1^−/−^ mice compared with WT mice as is the intestinal inflammation in basal conditions (Supplementary Fig. 1c, d).

### Microbiota composition is altered in PepT1^−/−^ mice

**Fig. 2:**
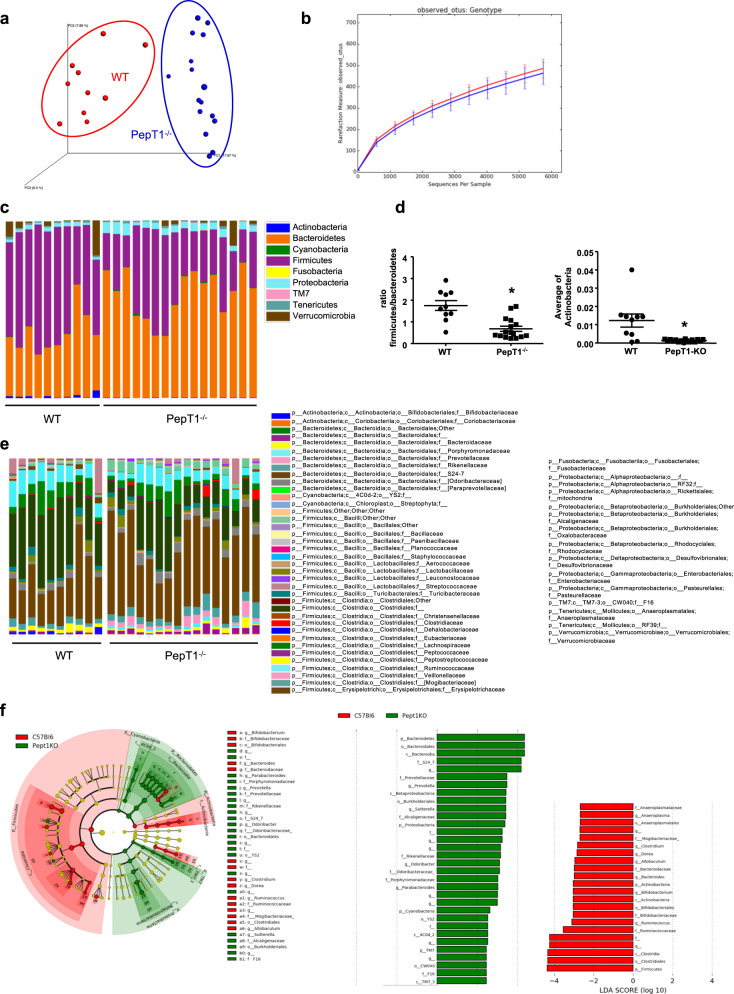
The microbiota composition of PepT1^−/−^ mice is altered. **a** Principal component analysis (PCA) of the unweighted UniFrac distance matrix of fecal WT and PepT1^−/−^ microbiota. **b** Microbiota richness and diversity in WT and PepT1^−/−^ mice, based on the number of OTUs per sample. **c** Bacterial-taxon–based analysis of feces at the phylum level. **d** Firmicutes/Bacteroidetes ratio and relative abundance of Actinobacteria. Bacterial-taxon–based analysis of feces at the family level (**e**). Data are presented as the mean ± SEM. Unpaired two-tailed Student’s *t*-test (*n* = 10–16 mice/group; *P < 0.05). **f** LEfSe (LDA Effect Size) was used to investigate bacterial members that drive the differences in the fecal microbiota of WT and PepT1^−/−^ mice. Left panel: Taxonomic cladogram obtained from LEfSe analysis of 16 S sequences. Red, WT taxa; green, PepT1^−/−^ taxa. The brightness of each dot is proportional to the size of its effect size. Right panel: LDA scores for the differentially altered taxa. Red, WT taxa; green, PepT1^−/−^ taxa. Only taxa meeting an LDA significance threshold >2.0 are represented.

Assessment of microbiota composition revealed an altered microbiota composition in PepT1^−/−^ mice compared with their WT counterpart, as observed by principal component analysis (Fig. 2a) and LEfSe analysis (Fig. 2f), although α-diversity was unchanged (Fig. 2b). A closer examination of composition at the phylum level revealed alterations in the abundance of some phyla that were similar to those previously described in other pathological conditions, such as obesity and IBD^22–24^ (Fig. 2c, d). For example, the abundance of Bacteroidetes was increased in PepT1^−/−^ mice, possibly contributing to the observed decrease in the Firmicutes/Bacteroidetes ratio, and the abundance of Actinobacteria was increased (Fig. 2d). Analysis at the family level shows that the differences described at the phylum level are mainly due to a decreased abundance of Clostridiales and an increased abundance of S24-7 in PepT1^−/−^ mice compared to WT mice (Fig. 2e). To more deeply characterize the microbiota alterations in Pept1^−/−^ mice, we have performed a multiple t-test analysis with a False Discovery Rate (FDR) approach at the OTU level, and identified 315 microbiota members (OTUs) that were significantly altered in PepT1^−/−^ mice compared with WT mice; of them, 101 OTUs were increased and 214 were decreased in PepT1^−/−^ mice (Supplementary Fig. 2). Interestingly, we observed decreases in the abundances of Akkermansia Muciniphila, Lactobacillus or Enterobacteriaceae and increases in the abundances of S24-7 or Rikenellaceae. A predictive functional analysis of the microbiota using PICRUSt illustrates that the changes in microbiota composition in PepT1^−/−^ mice are likely associated with changes in numerous pathways that could be involved in the protective phenotype observed in PepT1^−/−^ mice (Supplementary Fig. 3a). For example, the top 19 predicted deregulated biological functions included decreased bacterial toxins and glycosphingolipid biosynthesis, and increased protein digestion and cell division (Supplementary Fig. 3b). Decreased bacterial chemotaxis, flagellar assembly and bacterial motility were other functions that were altered in a way that could explain reduced penetrance of bacteria in the mucus in close contact with the intestinal epithelium (Supplementary Fig. 3c).

### The PepT1^−/−^ microbiota is sufficient to protect against colitis

**Fig. 3:**
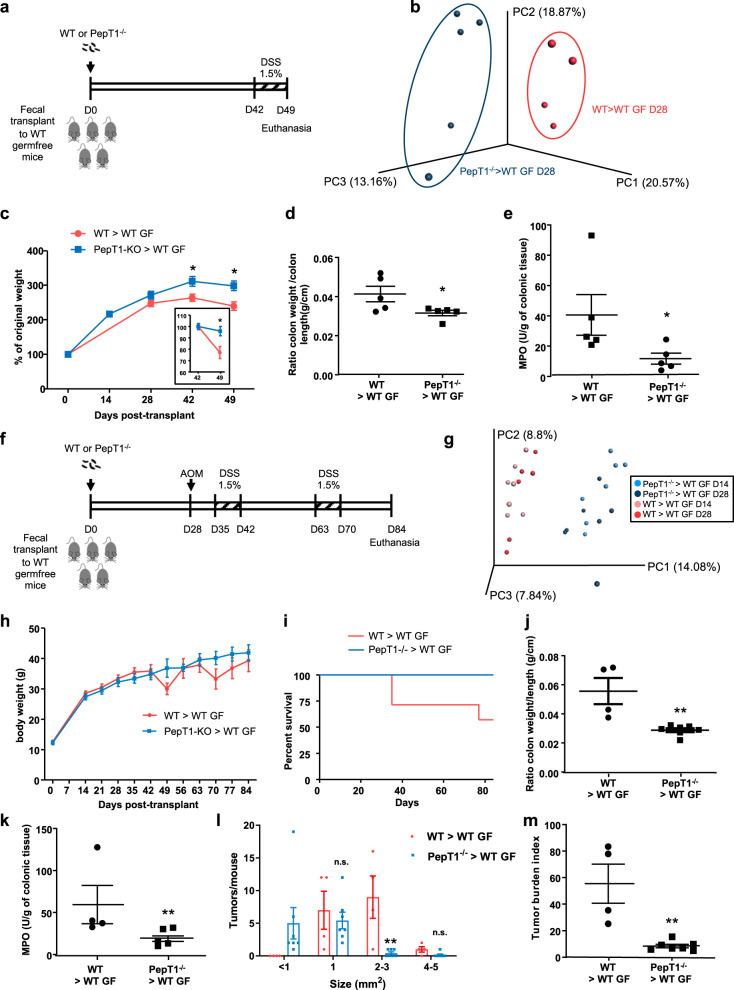
The microbiota of PepT1^−/−^ mice protects against colitis and CAC. **a**–**e** Three-week-old male GF C57BL/6 mice were conventionalized via microbiota transplant from a pool of two WT or PepT1^−/−^ female donor mice. Colitis was induced at D42 post transplantation by supplying 1.5% DSS in the drinking water. Mice were euthanized at D49 post transplantation. **a** Scheme of the experimental design. **b** Principal component analysis (PCA) of the weighted UniFrac distance matrix of fecal microbiota from WT (red) and PepT1^−/−^ (blue) microbiota recipient mice at D28 post-transplant. **c** Body weights of conventionalized mice from transplant to euthanasia at D49. **d** Colon weight/colon length ratio at D49. **e** MPO activity in the distal colon at D49. Significance was determined by unpaired two-tailed Student’s *t*-test (*n* = 5 mice/group; *P < 0.05). **f**–**m** Three-week-old female and male GF C57BL/6 mice were conventionalized via microbiota transplant from a pool of two WT or PepT1^−/−^ male donor mice. CAC was induced using an AOM/DSS protocol starting at D28 post transplantation. Mice were euthanized at D84 post transplantation. **f** Scheme of the experimental design. **g** Principal component analysis (PCA) of the weighted UniFrac distance matrix of fecal microbiota from WT (red) and PepT1^−/−^ (blue) microbiota recipient mice at D14 and D28 post-transplant. **h** Body weights of conventionalized mice from transplant to euthanasia at D84. **i** Survival rate. Mice were euthanized after losing 20% of their initial body weight. **j** Colon weight/colon length ratio at D84. **k** MPO activity in the distal colon at D84. **l** Number of tumors per mouse. **m** Tumor size, determined using a dissecting microscope. Significance was determined by unpaired two-tailed Student’s *t*-test (*n* = 5–7 mice/group; *P < 0.05, **P < 0.01, n.s. non-significant). Data are presented as the mean ± SEM.

To investigate whether the microbiota of PepT1^−/−^ mice is sufficient to confer protection against colitis, we transplanted PepT1^−/−^ or WT microbiota into WT germ-free (GF) recipient mice. At 42 days (D42) post transplantation, we induced mild colitis by treating with 1.5% DSS for 7 days (Fig. 3a), and then analyzed the resulting phenotype in recipient mice. Mucus layer thickness and bacteria-to-intestinal epithelium distance were measured in control mice groups that received WT or PepT1^−/−^ microbiota, but without a DSS challenge. WT mice that were recipients of the PepT1^−/−^ microbiota also exhibited an increase in mucus layer thickness and distance of bacteria from the intestinal epithelium (Supplementary Fig. 4a–c), showing that this feature is intrinsic to the microbiota and might be the result of imprinting of the host genotype on the microbiota. The transplanted WT and PepT1^−/−^ microbiota distinctly colonized recipient mice by day 28 (Fig. 3b). DSS treatment induced a greater degree of weight loss, measured on D42 post transplantation, in mice that received the WT microbiota compared with mice that receive the PepT1^−/−^ microbiota (Fig. 3c). The analysis of several parameters of inflammation, colon weight/colon length ratio (an indicator of overall colonic inflammation) and colonic myeloperoxidase (MPO) activity (a marker of tissue infiltration of neutrophils) as well as histological examination and scoring revealed a decrease of inflammation in mice transplanted with a WT microbiota (Fig. 3d, e and Supplementary Fig. 4d, e), indicating that colitis was more severe in mice receiving a WT microbiota than those receiving a PepT1^−/−^ microbiota. These results indicate that the altered microbiota of PepT1^−/−^ mice is sufficient to confer a level of protection against colitis compared with the microbiota of WT mice. That the phenotype of donor mice is observed in recipient mice in which genetics are held constant, also favors microbiome dominance over host gene dominance^25^.

### The PepT1^−/−^ microbiota is sufficient to protect against CAC

PepT1^−/−^ mice not only have a lower susceptibility to colitis but also to CAC^6^. Hence, we investigated if the microbiota of PepT1^−/−^ mice was also sufficient to protect against CAC. To this end, PepT1^−/−^ or WT microbiota were transplanted into WT GF mice. After allowing 28 days for the microbiota to stabilize (as seen Fig. 3b), we induced CAC using an AOM/DSS treatment protocol consisting of a single injection of AOM (azoxymethane) at D28 and two cycles of DSS, each consisting of 7 days of 1.5% DSS and 14 days of recovery (Fig. 3f). Two distinct microbiota colonized the recipient mice and remained stable over time, as evidenced by donor-type–specific microbiota compositions that were not significantly different between D28 and D14 (Fig. 3g). Mice that received either WT or PepT1^−/−^ microbiota had similar body weight when AOM/DSS protocol was initiated at Day 28. The two successive rounds of 7 days-DSS (starting at D49 and D63) induced a body weight loss in WT microbiota recipient mice that was not observed in mice receiving the PepT1^−/−^ microbiota (Fig. 3h), confirming a lower DSS sensitivity in mice harboring a PepT1^−/−^ microbiota. Mice experiencing weight loss greater than 20% of their maximal body weight were preemptively euthanized. Some mice receiving the WT microbiota had to be euthanized during the first DSS cycle as well as at the end of the second cycle, whereas none of the PepT1^−/−^ recipient mice lost more than 20% of their maximal body weight; as a result, survival rate was lower in mice that received the WT microbiota (Fig. 3i). The colon weight/colon length ratio, as well as colonic MPO activity, were lower in mice receiving the PepT1^−/−^ microbiota (Fig. 3j and k), indicating less inflammation in these mice compared with recipients of the WT microbiota. A subsequent investigation of tumorigenesis showed that mice that received the PepT1^−/−^ microbiota had a smaller number of larger tumors (≥2 mm^2^) and a lower overall tumor burden index (measured as total tumor surface area) compared with mice that received a WT microbiota, in which all tumors were >1 mm^2^ (Fig. 3l, m). These results suggest that the PepT1^−/−^ microbiota not only protects against colitis, but it also protects against consequent carcinogenesis.

### The protective effect of the PepT1^−/−^ microbiota is lost in separately housed littermates

**Fig. 4:**
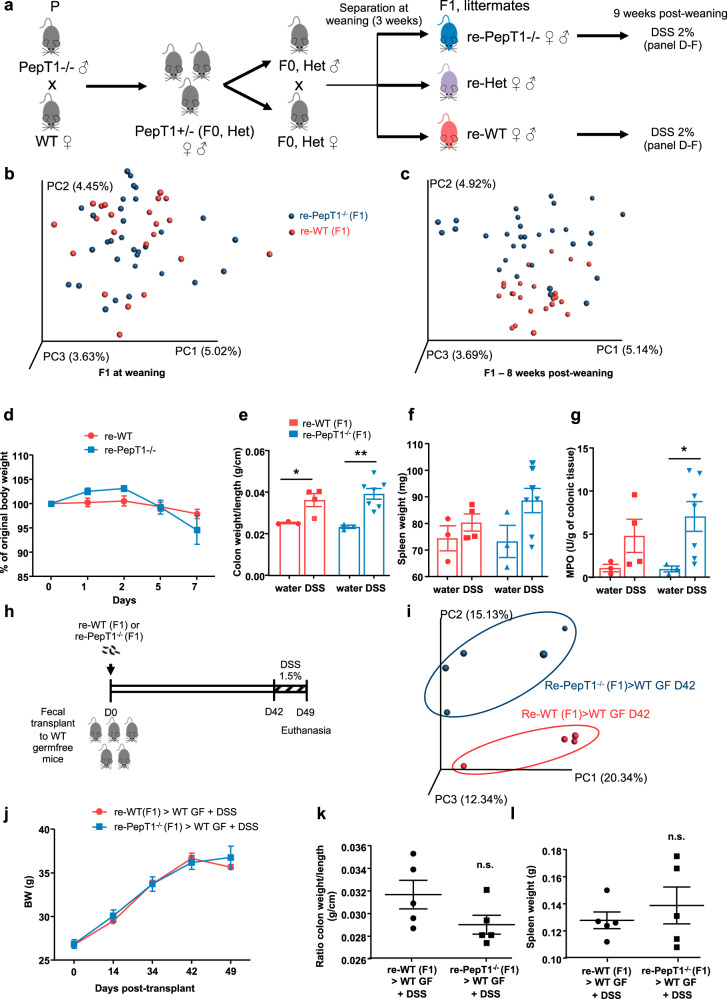
The microbiota of PepT1^−/−^ and PepT1^+/+^ true littermates does not differentially protect against DSS-induced colitis. **a** Breeding and housing scheme of WT and PepT1^−/−^ mouse experimental design. **b**, **c** Principal component analysis (PCA) of the weighted UniFrac distance matrix of fecal microbiota from re-WT (red) and re-PepT1^−/−^ (blue) mice at weaning (**b**) and 8 weeks post weaning (**c**). **d**–**g** Colitis was induced in F1 re-WT and re-PepT1^−/−^ littermates at 9 weeks post weaning by supplying 2% DSS in the drinking water. Mice were euthanized after 7 days of DSS (*n* = 4–7 mice/group). **d** Body weights during the DSS-treatment period. **e** Colon weight/colon length ratio. **f** Spleen weight. **g** MPO activity in the distal colon. Significance was determined by one-way ANOVA followed by a Bonferroni post-hoc test (*P < 0.05; **P < 0.01); **h**–**l** Three-week-old female GF C57BL/6 mice were conventionalized via microbiota transplant from a pool of three re-WT or re-PepT1^−/−^ donor mice. Colitis was induced at D42 post transplantation by supplying 2% DSS in the drinking water. Mice were euthanized at D49 post transplantation (*n* = 5 mice/group). **h** Scheme of the experimental design. **i** Principal component analysis (PCA) of the weighted UniFrac distance matrix of fecal microbiota from re-WT (red) and re-PepT1^−/−^ (blue) microbiota recipient mice at D42 post-transplant. **j** Body weights of conventionalized mice from transplant to euthanasia at D49. **k** Colon weight/colon length ratio. **l** Spleen weight. Significance was determined by unpaired two-tailed Student’s t test (n.s. non-significant). Data are presented as the mean ± SEM.

One logical explanation for the above-described results is that the PepT1-null genotype exerts a beneficial effect on the intestinal microbiota relative to a WT genotype. If so, we would expect to observe the same pattern of results when comparing PepT1^−/−^ and their WT littermates. To test this, we mated PepT1^−/−^ males with C57Bl/6 females (termed as P, parents) to generate PepT1^+/−^ (F0-het) mice, which after intercrossing yielded WT, PepT1^−/−^ and PepT1^+/^^−^ littermate offspring, termed re-WT F1, re-PepT1^−/−^ F1 and re-het F1, respectively. To allow for independent maturation of the microbiota in mice that experienced the same environmental exposures early in life, we housed re-WT and re-PepT1^−/−^ littermates separately at weaning (Fig. 4a). A principal component analysis at weaning showed that the fecal microbial population of co-housed littermates did not cluster according to PepT1 genotype (Fig. 4b). Similarly, re-PepT1^−/−^ mice and re-WT littermates physically separated at weaning showed no dramatic overall dysbiosis at 8 weeks post weaning (Fig. 4c). We compared the microbiota composition of the parents and littermates (re-WT and re-PepT1^−/−^ F1) of the PepT1^−/−^ and WT mice, and identified OTUs with significant differences of abundance between the parents and the PepT1^−/−^ and WT mice (separate breeders) (for a heatmap representation, see Supplementary Fig. 5a). Some OTUs were increased in PepT1^−/−^ mice (at the top of the heatmap), while others were decreased (bottom) (Supplementary Fig. 5a). On the same heatmap we plotted the values of relative abundance values of these OTUs in 8-week-old re-WT and re-PepT1^−/−^ true littermates (F1). Notably, the OTUs that differed significantly in abundance between WT and PepT1^−/−^ in the parent mice of separate breeders did not differ between re-WT and re-PepT1^−/−^ F1 littermates. Instead, the abundances of these OTUs were intermediary between the two genotypes in F1 mice, explaining why the inflammatory phenotype did not differ in the littermates (Supplementary Fig. 5a).

To determine whether re-PepT1^−/−^ mice were still protected against DSS-induced colitis compared with their re-WT littermates, we treated F1 re-WT and re-PepT1^−/−^ mice with 2% DSS 9 weeks after weaning. Weight loss (Fig. 4d), colon weight/colon length ratio (Fig. 4e), spleen weight (Fig. 4f) and colonic MPO activity (Fig. 4g), was not statistically different between DSS-treated WT and PepT1^−/−^ mice, suggesting that physical separation at weaning was not sufficient to affect the host susceptibility to colitis. To verify that this characteristic is conferred by the microbiota, we transplanted F1 re-PepT1^−/−^ or re-WT microbiota into WT GF mice (Fig. 4h). A principal component analysis indicated that the microbiota differed in the two groups of recipient mice (Fig. 4i). Taxa summarizations at the phylum and family levels (Supplementary Fig. 5b, c, respectively), reveal that the clear differences seen between parental WT and PepT1−/− microbiota in phyla Bacteroidetes and Firmicutes and family S24-7 and order Clostridiales (Fig. 2) were no longer present in the GF mice recipient of F1 littermate microbiota. LEfSe analysis shows that the few taxa differences observed between re-PepT1−/− and re-WT microbiota-recipient mice were mostly driven by Alphaproteobacteria, Cyanobacteria and Actinobacteria (Supplementary Fig. 5d).

The mice experienced similar weight loss regardless of whether they received re-PepT1^−/−^ or re-WT microbiota (Fig. 4j), and after euthanasia were found to have similar colon weight/colon length ratio (Fig. 4k) and spleen weight (Fig. 4l). There was no decrease in susceptibility to DSS-induced colitis in re-PepT1^−/−^ mice compared with their re-WT littermates, in contrast to the case of PepT1^−/−^ and WT mice bred separately for multiple generations.

### More than one generation is necessary for development of the protective phenotype in PepT1^−/−^ mice

**Fig. 5:**
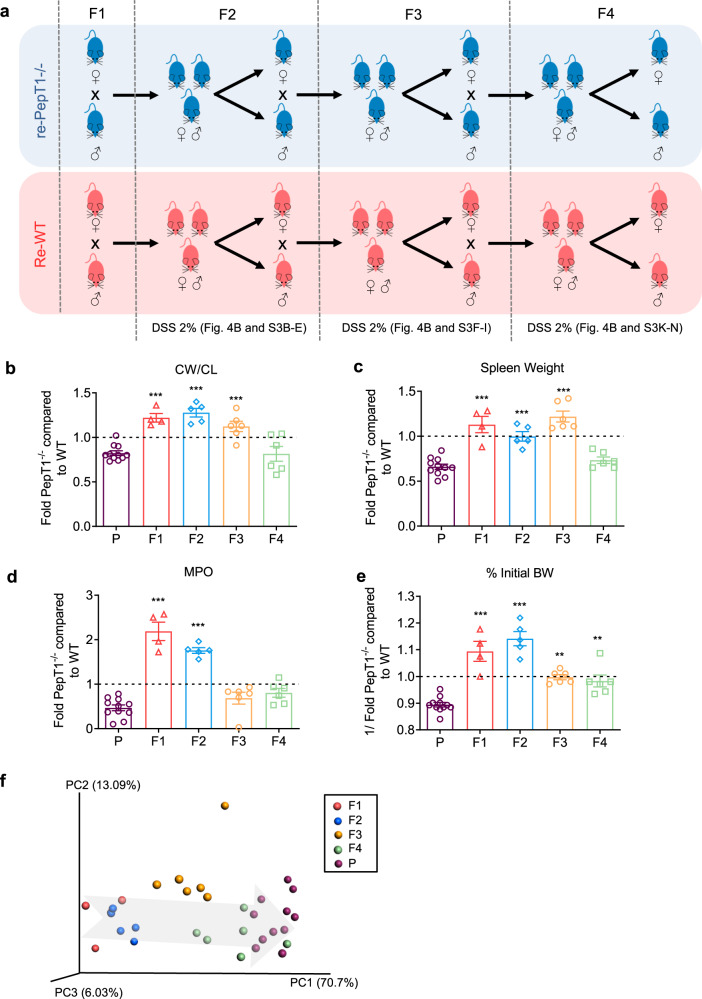
The protective phenotype of PepT1^−/−^ mice becomes established over generations. **a** Breeding and housing scheme of PepT1^+/-^ (Het, F0) used to obtain successive generations of re-WT and re-PepT1^−/−^ mice. Colitis was induced in re-WT and re-PepT1^−/−^ mice at 9 weeks post weaning by supplying 2% DSS in the drinking water. Mice were euthanized after 7 days of DSS (*n* = 4–11 mice/group). **b**–**e** Fold-change in parameters of inflammation in (re)-PepT1^−/−^ relative to (re)-WT across generations from parents (P) to generation F4. Fold-change in colon weight/colon length ratio (**b**), spleen weight (**c**), MPO activity in the distal colon (**d**), and 1/fold-change in body weight (**e**) after 7 days of DSS. Data are presented as the mean ± SEM. Significance was determined by one-way ANOVA followed by a Bonferroni post-hoc test compared to P (**P < 0.01; ***P < 0.001). **f** Principal component analysis (PCA) of the Bray Curtis distance of four inflammation parameters—colon weight/colon length ratio, spleen weight, colonic MPO and percentage of initial body weight after 7 days of DSS in PepT1^−/−^ versus WT—expressed as fold-change.

The results presented above using separately housed littermates indicate that physical separation after weaning does not allow the host PepT1 genetic status to influence the gut microbiota toward its protective characteristic. However, F1 re-WT and re-PepT1^−/−^ mice spent a 3-week period of co-housing from birth until weaning. Given that this pre-weaning period is an important window for establishing the gut microbiota^26,27^, we next evaluated the possibility that more than one generation was required to allow the protective phenotype to manifest. Accordingly, we established breeding pairs of homozygous re-WT and re-PepT1^−/−^ (F1) mice, and obtained F2, F3 and F4 generations, as described in Fig. 5a. We then subjected re-WT and re-PepT1^−/−^ mice from F2, F3 and F4 generations to a 2% DSS treatment protocol and compared their susceptibility to colitis. Following DSS treatment, F2 generation re-PepT1^−/−^ mice showed diminished weight loss (Supplementary Fig. 6a), a greater increase in colon weight/colon length ratio (Supplementary Fig. 6b) and colonic MPO activity (Supplementary Fig. 6d), and similar spleen weight (Supplementary Fig. 6c) compared with re-WT mice. At generation F3, re-PepT1^−/−^ and re-WT mice showed similar weight loss, but re-PepT1^−/−^ mice showed an increase in colon weight/colon length ratio, spleen weight and colonic MPO activity compared with re-WT mice (Supplementary Fig. 6e–h). At generation F4, whereas weight loss and increases in colonic MPO activity were similar in re-PepT1^−/−^ and re-WT mice following DSS treatment (Supplementary Fig. 6i,l), re-PepT1^−/−^ showed a smaller increase in colon weight/colon length ratio compared with re-WT mice and no increase in spleen weight (Supplementary Fig. 6j, k). To better visualize the progression of colitis susceptibility in re-PepT1^−/−^ mice compared with re-WT mice across generations from parents (P) to F4, we expressed the various parameters of inflammation as fold-difference in re-PepT1^−/−^ relative to re-WT and plotted all fold-changes for a given inflammatory parameter on the same graph and compared it to the parents (Fig. 5b–e). In the parents, as expected, the fold-difference in colon weight/colon length ratio (Fig. 5b), spleen weight (Fig. 5c) and colonic MPO (Fig. 5d), and the 1/fold-change ratio of the percentage of initial body weight (Fig. 5e) in PepT1^−/−^ compared with WT mice were less than 1, indicating that DSS-induced colitis was less severe in PepT1^−/−^ mice than in WT mice. At F1 and F2, however, fold-changes between PepT1^−/−^ and WT mice for all studied parameters are significantly increased compared to those of the parent generation, indicating that re-PepT1^−/−^ mice were no longer protected in F1 and F2 against DSS-induced colitis compared with re-WT mice. At generation F3, fold-changes in colon weight/colon length ratio, spleen weight and 1/fold-change in the percentage of the initial body weight were significantly increased compared to the parents, but colonic MPO was not; the latter parameter exhibited a fold-change in re-PepT1^−/−^ compared with re-WT that did not differ from that of the parents. At F4, all parameters except percentage of initial body weight were restored to the levels observed in parent mice (Fig. 5b–e). A principal component analysis of Bray Curtis distance using the same data showed that colitis susceptibility in re-PepT1^−/−^ mice across generations F1 to F4 returned to that in the parent generation, as evidenced by the fact that the F4 group mostly clustered with the parent (P) group (Fig. 5f). Taken together, these results suggest that the phenotype of re-PepT1^−/−^ mice comes to differ from that of re-WT mice as generations progress, approaching a lower susceptibility to DSS-induced colitis comparable to that in the parents (see also Figs 1 and 3).

### The PepT1^−/−^ microbiota establishes itself with the passage of generations

**Fig. 6:**
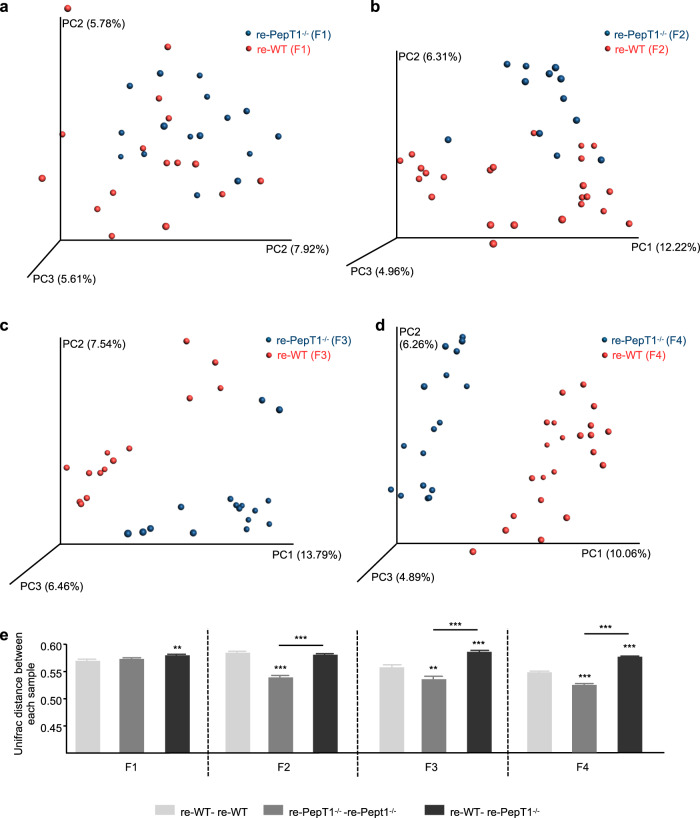
The microbiota evolves over generations. **a**–**d** Principal component analysis (PCA) of the unweighted UniFrac distance matrix of fecal re-WT and re-PepT1^−/−^ microbiota at generation F1 (**a**), F2 (**b**), F3 (**c**) and F4 (**d**). **e**, Average of unweighted UniFrac distances among re-WT (reWT-reWT), between re-WT and re-KO, and among re-KO at generation F1, F2, F3 and F4 (from left to right). Data are presented as the mean ± SEM. Significance was determined by one-way ANOVA followed by a Bonferroni post hoc test versus reWT-reWT or as indicated by the bars (**P < 0.01; ***P < 0.001).

To further investigate our hypothesis that the protection from/susceptibility to colitis of PepT1^−/−^ mice compared with WT mice is microbiota related, we next analyzed the microbiota composition across generations at 8 weeks post-weaning (Fig. 6). We found that the microbiota at F1 was similar between re-WT and re-PepT1^−/−^ mice (Fig. 6a); at F2, the microbiota of re-WT and re-PepT1^−/−^ slightly diverged (Fig. 6b); and at F3 and F4, the microbiota composition of the two groups of mice differed (Fig. 6c and d). A deeper microbiota analysis at each generation from F1 to F4 is shown Supplementary Fig. 7. Taxa summarization at the phylum level and Lefse analysis show that phylum ratios are progressively changing over generations. OTU tables at the family level detailed the taxonomic changes at the family level from generation F1 to generation F4 and show that the abundance of a larger number of families were altered in re- PepT1^−/−^ as generation progressed with for example, modified abundance of S24-7 or Rikenellacea families at generation F3 and F4 that were no longer seen in F1 and F2 (Supplementary Data 1–4). We then calculated the UniFrac distance among re-WT samples, among re-PepT1^−/−^ samples and between re-WT and re-PepT1^−/−^ samples from generation F1 to F4 (Fig. 6e). We found that the distance between re-WT and re-PepT1^−/−^ became higher across generations, while remaining similar among re-WT samples and lower among re-PepT1^−/−^ samples as generations progressed (Fig. 6e). These results indicate that from generation F2 on, the microbiota of re-WT and re-PepT1^−/−^ mice differed and stabilized toward WT and PepT1^−/−^ microbiotas, respectively, whereas at F1, the microbiotas of re-WT and re-PepT1^−/−^ littermates were largely the same. The phenotype and microbiota composition of F4 PepT1^−/−^ mice indicate that the microbiota evolves across generations toward equilibrium in the context of a genetic alteration. To understand whether the abundance of certain bacteria could explain variations in the susceptibility to DSS-induced colitis in WT and PepT1^−/−^ mice, we correlated the abundance of operational taxonomic units (OTUs), a measure of taxonomic clustering, to parameters of inflammation obtained from WT and PepT1^−/−^ mice with DSS-induced colitis (Supplementary Fig. 8). This analysis revealed significant correlations, indicating that the degree of inflammation, as assessed by colon weight/colon length ratio (Supplementary Fig. 8a), spleen weight (Supplementary Fig. 8b), percent of initial body weight (Supplementary Fig. 8c) and colonic MPO activity (Supplementary Fig. 8d), correlated with the abundance of some bacteria at the genus level. Taken together, these results reveal a microbiota that becomes established over generations and correlates with the evolving inflammatory phenotype across generations.

## Discussion

We previously reported that mice with a genetic deletion of PepT1 have decreased susceptibility to DSS-colitis and CAC^5,6^. Here, we report that this deletion has drastic consequences on the composition and localization of the intestinal microbiota, leading to a more beneficial state that promotes reduced susceptibility to induced colitis. In PepT1^−/−^ mice, we observed changes in the localization of bacteria, a thicker mucus layer and increased numbers of goblet cells per crypt—all of which can influence the susceptibility of mice to induced colitis—as well as a decreased Bacteroidetes/Firmicutes ratio and a decreased abundance of Actinobacteria. Fecal transplant experiments identified the microbiota as crucial to the protective phenotype observed in PepT1^−/−^ mice. However, these results were obtained comparing WT and PepT1^−/−^ that had not been bred or had other direct contact for many generations thus suggesting that the difference may not reflect genotype per se.

Indeed, recent studies on the NLRP6 inflammasome by Girardin, Philpot and McCoy and Wullaert groups suggested that the use of littermates is the ‘gold standard’ approach for studying the impact of a mutation (genetic variable) on the microbiota or a disease^13–15^. Non-genetic variables could affect the microbiota of genetically identical mice, leaving open the possibility that our phenotype and the alterations of the microbiota observed in our non-littermate PepT1^−/−^ mice occurred randomly. It has also been suggested that the most scientifically sound method for studying microbiota is to compare genetically modified mice to their WT littermates^15^. Hence, we generated WT and PepT1^−/−^ littermates and observed that re-PepT1^−/−^ (F1) mice no longer had a lower susceptibility to DSS-induced colitis than re-WT (F1) mice suggesting our initial results were artifactual. For example, our previous study suggested that depletion of PepT1 in immune cells was playing an important role in colitis and is dependent on the microbiota^5^. At first, this conclusion could seem to be invalidated in the current study as we observed no difference between re-WT (F1) and re-PepT1^−/−^ (F1) with regard to colitis. However, further investigation found that, in fact, alterations in microbiota and colitis susceptibility PepT1^−/−^ is a direct consequence of genotype but requires multiple generations to emerge.

The pre-weaning period is critical in shaping the microbiota^27^. We thus established homozygous breeding pairs of re-WT and re-PepT1^−/−^ mice and afforded them the opportunity to shape their microbiota across four generations. We observed a progressive return of the protective microbiota in PepT1^−/−^ mice across generations, confirming that the PepT1^−/−^ microbiota protects against colitis and CAC. We also identified correlations between specific bacteria and parameters of inflammation. Our results thus indicate that effects of a given genotype on the microbiota will not necessarily be observed in a true littermate experiment; in some cases, establishment of a genotype dependent-phenotype may require separation and multiple generations of separate crossings. In their studies, Lemire and Mamantopoulos argued that if a post-weaning separation causes no shift in the microbiota, then we can infer a non-genotype–dependent effect^13,14^, casting doubt on Elinav’s finding that the NLRP6 inflammasome regulates the gut microbiota and colitis^28^. However, our data on another genetic deletion suggest otherwise, arguing that in some cases multiple generations could be necessary for the genotype-dependent microbiota to reach its steady state. Depending on the nature of the mutation, we can imagine that a phenotype could either become established in littermates or after several generations of consistent crossing.

In humans, one of the strongest susceptibility genes associated with a post-weaning separation is NOD2 (nucleotide binding oligomerization domain containing 2), in which a frame shift mutation was identified in Crohn’s disease (CD) families^29,30^. Yet, an essential role for NOD2 in the temporal development and composition of the host microbiota was identified in both mice and humans that may contribute to the complex role of NOD2 in the etiopathogenesis of CD^31^. In this latter study, the authors backcrossed *Nod2*^−/−^ breeding pairs against the C57BL/6 J strain, and then selected *Nod2*^+/+^ and *Nod2*^−/−^ littermates to start new homozygous intercrossed lines that were housed separately. The mutation substantially altered fecal microbiota composition and bacterial load in *Nod2*^−/−^ mice compared with their WT counterparts. The same findings were also observed in weaning mice, indicating a profound influence of NOD2 on the early development and composition of the intestinal microbiota, and corroborating findings in humans^31^. Our study failed to detect host genetic effects in PepT1^−/−^ F1 littermates, as was the case for other studies on the NLRP6-inflammasome^13,14^. In studies investigating other mutations, for example using the epithelial-specific TLR (toll-like receptor) 5-deficient mice or Card9 (caspase recruitment domain family, member 9)-deficient mice^32,33^, the authors observed a genotype effect on the gut microbiota in as little as 4–6 weeks. The former observations do not argue that there is no genotype-driven effect if no phenotype is observed within this time frame, but rather suggest that the influence of a mutation on the microbiota is variable and depends on the type of mutation. Taken together, these studies suggest that, depending on the mutation, different approaches may be necessary to decipher the degree of influence of the genotype on the microbiota and/or phenotype.

## Methods

### Mice

Male and female eight-week-old PepT1^−/−^ mice and their matched C57Bl/6 were used in this study. Mice were housed in specific pathogen-free conditions and fed ad libitum. PepT1^−/−^ males (P, parents) were mated with C57Bl/6 (P) females to generate PepT1^+/−^ (F0, het) mice (Fig. 4a). Littermate offsprings WT (re-WT F1), PepT1^−/−^ (re-PepT1^−/−^ F1) and PepT1^+/−^ (re-het F1) were obtained by intercrossing of the het (Fig. 4a). Re-WT and re-PepT1^−/−^ littermates were separately housed at weaning to allow for independent maturation of microbiota in mice. Re-WT and re-PepT1^−/−^ (F1) were used to set up homozygous breeding pairs and generation F2, F3 and F4 were obtained as described on Fig. 5a. re-WT and re-PepT1^−/−^ were separately housed at weaning (3 weeks of age). Moderate colitis was induced in female and male 11 week-mice (8 weeks post-weaning) from generation F1, F2, F3 and F4 by using 2% DSS for 7 days. Mice were euthanized after the 7 days of DSS treatment and tissues were collected for analysis. Feces of each generation of mice were collected at weaning and at 8 weeks of age (before starting the DSS treatment) for microbiota analysis. All the experiments involving mice complied with ethical regulations for animal testing and research and were approved by the institutional animal care and use committee (IACUC, Georgia State University Atlanta, GA, USA, #A17044).

### Germ-free experiments

Germ-free C57Bl/6 or Swiss Webster mice were kept under germfree conditions in a Park Bioservices isolator in our germ-free facility. Conventional age-matched and sex-matched C57Bl/6 or Swiss Webster mice were used in parallel.

### Microbiota transplantation

Fecal contents from C57Bl/6 WT or PepT^1−/−^ mice were suspended in 30% glycerol diluted in PBS (1.0 ml) and stocked at −80°C until analysis. Germ-free C57Bl/6 or Swiss Webster mice (4 weeks old) were removed from the isolator and, at Day 0, were orally administered 200 µl of fecal suspension made using glycerol stocks. Either mild colitis or colitis-associated cancer was induced in the recipient mice with respectively, 1.5% DSS from Day 42 to Day 49 post-transplantation (as schematized on Fig. 3a) or AOM/DSS protocol from Day 28 to Day 84 post-transplantation (as schematized on Fig. 3f). Feces were collected at D14 and D28 post-transplantation in order to analyze the microbiota in the recipient mice. Mice from the two experimental protocols were respectively euthanized at Day 49 (colitis) or Day 84 post-transplantation (colitis-associated cancer) and tissues collected for analysis.

### H&E staining of colonic tissue and histopathologic analysis

Mouse colons were fixed in 10% buffered formalin for 24 h at room temperature and then embedded in paraffin. Tissues were sectioned at 5-μm thickness and stained with hematoxylin & eosin (H&E) using standard protocols. Images were acquired using a Zeiss Axioskop 2 plus microscope (Carl Zeiss MicroImaging) equipped with an AxioCam MRc5 CCD camera (Carl Zeiss). Histological scoring was blindly determined on colon^34,35^. Each colon was assigned four scores based on the degree of epithelial damage [0 = normal; 1 = hyperproliferation, irregular crypts, and goblet cell loss; 2 = mild to moderate crypt loss (10–50%); 3 = severe crypt loss (50–90%); 4 = complete crypt loss, surface epithelium intact; 5 = small- to medium-sized ulcer (<10 crypt widths); 6 = large ulcer (≥10 crypt widths)] and inflammatory infiltrate in the mucosa (0 = normal, 1 = mild, 2 = modest, 3 = severe), submucosa (0 = normal, 1 = mild to modest, 2 = severe) and muscularis/serosa (0 = normal, 1 = moderate to severe)^36^. Each of the four scores was multiplied by a coefficient of 1 if the change was focal, 2 if it was patchy and 3 if it was diffuse^34^, and the four individual scores per colon were summed. The summed scores of all mice of each treatment group were averaged.

### Bacterial quantification by qPCR

For quantification of total fecal bacterial load, total bacterial DNA was isolated from weighted feces using QIAamp DNA Stool Mini Kit (Qiagen) after a step of mechanical disruption (bead beating). See supplementary methods for details.

### Fecal lysozyme activity

Fecal proteins were mechanically extracted after rehydration of frozen feces in 500 ml PBS (10 mM; pH 7.2). After 10 min of centrifugation at 1600g, supernatants were sterilized with a 0.22-mm filter and frozen. Fecal protein concentration was measured using DC assay (BioRad). Activity of lysozymes against the peptidoglycan was determined using the EnzChek Lysozyme Assay Kit (Life Technology).

### Colonic mRNA expression analysis by qPCR

RNA Extraction and Real-Time RT-PCR Total RNA were extracted from colonic tissues using RNeasy mini Kit (Qiagen) according to the manufacturer’s instructions. See supplementary methods for details.

### Colonic myeloperoxidase (MPO) assay

See supplementary material and methods.

### Fecal flagellin and LPS load quantification

Flagellin and LPS were quantified using human embryonic kidney (HEK)-Blue-mTLR5 and HEK- BluemTLR4 cells, respectively (Invivogen)^37^. See supplementary material and methods for details.

### Immunostaining of mucins and localization of bacteria by fluorescent in situ hybridization

Mucus immunostaining was paired with fluorescent in situ hybridization, to analyze bacteria localization at the surface of the intestinal mucosa^38^. See supplementary material and method for details.

### Fecal microbiota analysis by 16S rRNA gene sequencing

16S rRNA gene amplification and sequencing were done using the Illumina MiSeq technology following the protocol of Earth Microbiome Project (http://www.earthmicrobiome.org/emp-standard-protocols)^39,40^. Bulk DNA were extracted from frozen extruded feces using a PowerFecal-htp kit with mechanical disruption (bead beating). See supplementary methods for details.

### 16S rRNA gene sequence analysis and metagenome prediction

The sequences were demultiplexed, quality filtered using the Quantitative Insights Into Microbial Ecology (QIIME, version 1.8.0) software package^41^, and forward and reverse Illumina reads were joined using the fastq-join method (https://expressionanalysis.github.io/ea-utils/)^42^. We used QIIME default parameters for quality filtering (reads truncated at first low-quality base and excluded if: (1) there were more than three consecutive low quality base calls; (2) less than 75% of read length was consecutive high quality base calls; (3) at least one uncalled base was present; (4) more than 1.5 errors were present in the barcode; (5) any Phred qualities were below 20; or (6) the length was less than 75 bases). Sequences were assigned to OTUs using the UCLUST algorithm^43^ with a 97% threshold of pairwise identity and with the creation of new clusters with sequences that did not match the reference sequences. OTUs were taxonomically classified using the Greengenes reference database^44^. A single representative sequence for each OTU was aligned and a phylogenetic tree was built using FastTree^45^. The phylogenetic tree was used for computing the unweighted UniFrac distances between samples^46,47^. Rarefied OTU table were used to compare abundances of OTUs across samples. Principal component analysis (PCA) plots were used to assess the variation between experimental group (beta diversity), alpha diversity curves were determined for all samples using the determination of the number of observed species, and OTU table was rarefied at various taxonomic levels using QIIME. Data were also represented as heatmap using Morpheus^48^ and taxa summarization. LEfSE (LDA Effect Size) was used to investigate bacterial members that drive differences between groups^49^. PICRUSt (Phylogenetic Investigation of Communities by Reconstruction of Unobserved States) was used to predict the metagenome based on microbiota composition analysis^50^ and Kyoto Encyclopedia of genes and genomes (KEGG) modules with an altered abundance in PepT1^−/−^ compared to WT were represented on a heatmap.

### Statistical analysis

Measurements were taken from distinct samples. Prior to determining the significance with parametric tests, normality was tested using D’Agostino & Pearson omnibus test. For normally distributed samples, significance was determined using either unpaired two-tailed Student’s *t*-test or one-way ANOVA followed by a Bonferroni post-test. Mann–Whitney test or Kruskal–Wallis test followed by a Dunn’s post-test were used when samples failed the normality test (GraphPad Prism 8 software). A F-test was used to determine the deviation from zero of the linear regression presented in Fig. S8. Histograms are showing means ± standard error of the mean (SEM) in order to report the precision of the mean. Differences were noted as significant *P < 0.05, **P < 0.01 and ***P < 0.001.

### Reporting summary

Further information on experimental design is available in the Nature Research Reporting Summary linked to this paper.

## Supplementary information

Supplementary Information

Supplementary Data 1

Supplementary Data 2

Supplementary Data 3

Supplementary Data 4

Reporting Summary

## Data Availability

Unprocessed sequencing data are deposited in the European Nucleotide Archive under accession number PRJEB38890. The authors declare that all other data supporting the findings of this study are available within the paper and its supplementary information files.
