# Supplementary material for: Impact of PepT1 deletion on microbiota composition and colitis requires multiple generations
Source: NPJ Biofilms Microbiomes. 2020 Jul 21;6:27. doi: 10.1038/s41522-020-0137-y (PMC7374158; doi:10.1038/s41522-020-0137-y)
Supplement: Supplementary file 1 — Supplementary Information [file 41522_2020_137_MOESM1_ESM.pdf]

SUPPLEMENTARY METHODS

Bacterial quantification by qPCR

For quantification of total fecal bacterial load, total bacterial DNA was isolated from weighted feces using QIAamp DNA Stool Mini Kit (Qiagen) after a step of mechanical disruption (bead beating). DNA was then subjected to quantitative PCR using QuantiFast SYBR Green PCR kit (Biorad) with universal 16SrRNAprimers 8F: 5'-AGAGTTTGATCCTGGCTCAG-3' and 338R: 5'-CTGCTGCCTCCCGTAGGAGT-3' to measure total bacteria. Results are expressed as number of 16S rRNA copy per mg of feces, using a standard curve. For quantification of mucosa-associated bacteria, total DNA was isolated from PBS-washed and weighted colonic tissue using DNeasy Blood & Tissue Kit (Qiagen) after a step of mechanical disruption (bead beating). DNA was then subjected to quantitative PCR as described above, and results are expressed as number of 16S rRNA copy per mg of tissue, using a standard curve.

Colonic mRNA expression analysis by qPCR

RNA Extraction and Real-Time RT-PCR Total RNA were extracted from colonic tissues using RNeasy mini Kit (Qiagen) according to the manufacturer's instructions. Yield and quality of RNA were verified with a Synergy 2 plate reader (BioTek, Winooski, VT, USA). cDNA was generated from the total RNA isolated above using the Maxima first-strand cDNA synthesis kit (Thermo Scientific, Lafayette, CO, USA). mRNA expression was quantified by quantitative real-time reverse transcription-PCR (qRT-PCR) using Maxima SYBR green quantitative PCR (qPCR) Master Mix (Thermo Scientific) and the following sense and antisense primers: Zg16-fw 5'-CTTCCGTATCCGGGTCAACA-3' and Zg16-rv 5'-ACAGCGTTGAAACTTGTGCC-3'; Lys1-fw 5'-GCCAAGGTCTACAATCGTTGTGAGTTG-3' and Lys1-rv 5'-

CAGTCAGCCAGCTTGACACCACG-3'; Defb1-fw: 5'-TCCTGGTGATGATATGTTTTCTTTTCT-3' and Defb1-rv: 5'-TGTTCTTCGTCCAAGACTTGTGA-3'; 36B4 5'-TCCAGGCTTTGGGCATCA-3' and 5'-CTTTATCAGCTGCACATCACTCAGA-3'. Results were normalized by using 36B4 housekeeping gene.

Colonic myeloperoxidase (MPO) assay

Colon tissues (50 mg/mL) were thoroughly washed in PBS and homogenized in pre-chilled 0.5% hexadecyltrimethylammonium bromide (Sigma, St. Louis, MO) in 50 mM PBS, (pH 6.0), freeze-thawed 3 times, sonicated and centrifuged. MPO was assayed in the clear supernatant by adding 1 mg/mL of dianisidine dihydrochloride (Sigma, St. Louis, MO) and $5x10^{-4}$% H2O2 and the change in absorbance was measured at 450 nm. Human neutrophil MPO (Sigma, St. Louis, MO) was used as standard. One unit of MPO activity was defined as the amount that degraded 1.0 µmol of peroxide per minute at 25°C.

Fecal flagellin and LPS load quantification

Flagellin and LPS were quantified as previously described [37] using human embryonic kidney (HEK)-Blue-mTLR5 and HEK- BluemTLR4 cells, respectively (Invivogen). Briefly, fecal material was resuspended in PBS to a final concentration of 100 mg/mL and homogenized using a Mini-Beadbeater-24 without the addition of beads to avoid bacteria disruption. Supernatants were serially diluted and applied to mammalian cells. Purified flagellin from *Salmonella typhimurium* and LPS from *Escherichia coli* (Sigma) were used for standard curve determination. After 24 hours of stimulation, cell culture supernatants were applied to QUANTI-Blue medium

(Invivogen) and alkaline phosphatase activity was measured at 620 nm after 30 minutes.

Immunostaining of Mucins and Localization of Bacteria by Fluorescent In Situ Hybridization

Mucus immunostaining was paired with fluorescent in situ hybridization, as previously described [38], to analyze bacteria localization at the surface of the intestinal mucosa. Colonic tissues containing fecal material were placed in methanol-Carnoy's fixative solution (60% methanol, 30% chloroform, 10% glacial acetic acid) for a minimum of 3 hours at room temperature. The tissues were then washed in methanol twice for 30 minutes, ethanol twice for 15 minutes, ethanol/xylene (1:1) for 15 minutes, and xylene twice for 15 minutes, followed by embedding in paraffin with a vertical orientation. Five-micrometer sections were obtained and dewaxed by preheating at 60°C for 10 minutes, followed by xylene at 60C for 10 minutes, xylene for 10 minutes, and 99.5% ethanol for 10 minutes. The hybridization step was performed at 50°C overnight with an EUB338 probe (5'-GCTGCCTCCCGTAGGAGT-3', with a 50 labeling using Alexa 647) diluted to a final concentration of 10 mg/mL in hybridization buffer (20 mmol/L Tris HCl, pH 7.4, 0.9 mol/L NaCl, 0.1% sodium dodecyl sulfate, 20% formamide). After washing for 10 minutes in wash buffer (20 mmol/L Tris HCl, pH 7.4, 0.9 mol/L NaCl) and 3 times for 10 minutes in PBS, PAP pen (Sigma Chemical Co, St Louis, MO) was used to mark around the section and block solution (5% fetal bovine serum in PBS) was added for 30 minutes at 4°C. Mucin-2 primary antibody (rabbit H-300; Santa Cruz Biotechnology, Santa Cruz, CA) was diluted 1:1500 in block solution and apply overnight at 4°C. After washing 3 times for 10 minutes in PBS, block solution containing anti-rabbit Alexa 488 secondary antibody diluted 1:1500, Phalloidin-Tetramethylrhodamine B isothiocyanate (Sigma) at 1 mg/mL, and Hoechst 33258 (Sigma) at 10 mg/mL was applied to the

section for 2 hours. After washing 3 times for 10 minutes in PBS, slides were mounted by using ProLong antifade mounting media (Life Technologies, Carlsbad, CA). Observations were performed with a Zeiss (Oberkochen, Germany) LSM 700 confocal microscope with software Zen 2011 version 7.1. This software was used to determine the distance between bacteria and the epithelial cell monolayer as well as the mucus thickness.

Fecal microbiota analysis by 16S rRNA gene sequencing

16S rRNA gene amplification and sequencing were done using the Illumina MiSeq technology following the protocol of Earth Microbiome Project (http://www.earthmicrobiome.org/emp-standard-protocols) [39, 40]. Bulk DNA were extracted from frozen extruded feces using a PowerFecal-htp kit with mechanical disruption (bead beating). The 16S rRNA genes, region V4, were PCR amplified from each sample using a composite forward primer and a reverse primer containing a 12-base barcode, which was used to tag PCR products from respective samples [3]. Forward primer 515F 5’-*AATGATACGGCGACCACCGAGATCTACAC***TATGGTAATT*****GT**GTGCCAGCMGCCGCGGTAA-3’ was used. The sequence in italic is the 5’ Illumina adaptor B and the sequence in bold is the primer pad. The sequence in both italic and bold (GT) is the primer linker. The underlined sequence is the conserved bacterial primer 515F. The reverse primer 806R 5’-*CAAGCAGAAGACGGCATACGAGAT* XXXXXXXXXXXX **AGTCAGTCAG** ***CC*** GGACTACHVGGGTWTCTAAT-3’ was used. The sequence in italic is the 3’ reverse complement sequence of Illumina adaptor, the 12X sequence is the Golay barcode, the sequence in bold is the primer pad, and the italicized and bold sequence is the primer linker. The underlined sequence is the conserved bacterial primer 806R. PCR reactions consisted of Hot Master PCR mix

(Five Prime), 0.2 mM of each primer, 10–100 ng template, and reaction conditions were 3min at 95°C, followed by 30 cycles of 45s at 95°C, 60s at 50°C and 90s at 72 °C on a Biorad thermocycler. Four independent PCRs were performed for each sample, combined, purified with Ampure magnetic purification beads (Agencourt), and products were visualized by gel electrophoresis. Products were then quantified (BIOTEK Fluorescence Spectrophotometer) using a Quant-iT PicoGreen dsDNA assay. A master DNA pool was generated from the purified products in equimolar ratios. The pooled products were quantified using a Quant-iT PicoGreen dsDNA assay and then sequenced using an Illumina MiSeq sequencer (paired-end reads, 2 X 250 base pairs) at Cornell University, Ithaca.

SUPPLEMENTARY FIGURE LEGENDS

Supplementary Figure 1: Goblet cells and proinflammatory potential in WT and PepT1$^{-/-}$ mice. Colon tissues and feces were collected from 6-month-old PepT1$^{-/-}$ and WT mice. **a,** Goblet cell staining with periodic acid and Alcian blue. Scale bar: 50 µm. **b,** Number of goblet cells per crypt in WT and PepT1$^{-/-}$ mice colonic tissues. **c-d,** Levels of bioactive fecal LPS **(c)** and flagellin **(d)** were assayed using TLR5 and TLR4 reporter cells, respectively. Data are presented as the mean ± SEM. Unpaired two-tailed Student's t test (n= 10–16 mice/group; **P < 0.01, n.s. non-significant).

Supplementary Figure 2: Microbiota composition in WT and PepT1$^{-/-}$ mice. Heatmap representation of OTU with an altered abundance in PepT1$^{-/-}$ compared with WT mice. A multiple t-test analysis with a False Discovery Rate (FDR) approach (two-stage step-up method of Benjamini, Krieger and Yekutieli) at the OTU levels was used. Abundance data are presented as

relative values ranging from low (blue) to high (red) expression. Hierarchical clustering of OTU (rows) and samples (columns) was performed using One Minus Pearson's correlation.

Supplementary Figure 3: Predicted metagenome in WT and PepT1$^{-/-}$ mice. PICRUSt (Phylogenetic Investigation of Communities by Reconstruction of Unobserved States) was used to predict the metagenomes of WT and PepT1$^{-/-}$ mice under basal conditions. Predicted metagenomes were categorized at level 3 of the Kyoto Encyclopedia of genes and genomes (KEGG) pathways. **a,** Heatmap representation of pathways with an altered abundance in PepT1$^{-/-}$ compared with WT mice. Expression data are presented as relative values, from low (blue) to high (red) expression. Hierarchical clustering of pathways (rows) and samples (columns) was performed using One Minus Pearson's correlation. **b,** Top 19 significantly altered pathways. **c,** Other altered pathways of interest. Data are presented as the mean ± SEM. Unpaired two-tailed Student's t test (n = 10–16 mice/group; **P < 0.01; ***P< 0.001).

Supplementary Figure 4: Microbiota localization in WT germ-free mice conventionalized with a WT or PepT1$^{-/-}$ microbiota. Three-week-old male GF C57BL/6 mice were conventionalized via microbiota transplant from a pool of two WT or PepT1$^{-/-}$ female donor mice. At 21 days post-transplantation, mice were euthanized and colon tissues were collected. **a,** Representative images obtained from confocal microscopy analyses of microbiota localization in Carnoy-fixed colonic tissues of WT and PepT1$^{-/-}$ microbiota recipient mice. **B** and **c**, The mucus thickness **(b)** and distance of the closest bacteria to the intestinal epithelium **(c)** were calculated across five high-powered fields. Green, Muc2; purple, actin; red, bacteria; blue, DNA. Scale bar: 20 µm. **d,** At 42 days post-transplantation, colitis was induced in WT mice conventionalized with

WT or PepT1$^{-/-}$ microbiota by supplying 1.5% DSS in the drinking water. Mice were euthanized at D49 post transplantation. Colon samples were paraffin-embedded, sectioned and stained with H&E. Scale bar: 1 mm. **e,** Histological score. Data are presented as the mean ± SEM. Significance was determined by unpaired two-tailed Student's t test (n = 5 or 6 mice/group; *P < 0.05; **P < 0.01.

Supplementary Figure 5: Microbiota compositions of PepT1$^{-/-}$ and PepT1$^{+/+}$ mice derived from separate breeders as well as that of the F1 littermates. a, OTUs whose abundance was significantly altered (multiple t-test analysis with a False Discovery Rate according the two-stage step-up method of Benjamini, Krieger and Yekutieli) altered between PepT1$^{-/-}$ and WT mice from separate breeders were selected and plotted in a heatmap, together with the relative abundance of the same OTUs in 8-week-old re-WT and re-PepT1$^{-/-}$ true littermates (F1). Abundance are represented as relative values ranging from low (blue) to high (red) expression. Hierarchical clustering of OTU (rows) was performed using One Minus Pearson's correlation. **b** and **c,** Bacterial-taxon–based analysis of fecal microbiota at the phylum **(b)** and family **(c)** level. **d,** LEfSe (LDA Effect Size) was used to investigate bacterial members that drive the differences in the fecal microbiota of re-WT (F1) and re-PepT1$^{-/-}$ (F1) mice. Upper panel: Taxonomic cladogram obtained from LEfSe analysis of 16S rRNA sequences. Red, WT taxa; green, PepT1$^{-/-}$ taxa. The brightness of each dot is proportional to the size of its effect size. Lower panel: LDA scores for the differentially altered taxa. Red, WT taxa; green, PepT1$^{-/-}$ taxa. Only taxa meeting an LDA significance threshold > 2.0 are represented.

Supplementary Figure 6: The protective phenotype of PepT1$^{-/-}$ mice across generations. Colitis was induced in re-WT and re-PepT1$^{-/-}$ mice at 9 weeks post weaning by supplying 2% DSS in the drinking water. Mice were euthanized after 7 days of DSS. **a,** Body weights of F2 re-WT and re-PepT1$^{-/-}$ mice during the DSS-treatment period. **b**, Colon weight/colon length ratio of F2 re-WT and re-PepT1$^{-/-}$ mice. **c,** Spleen weight at F2. **d,** MPO activity in the distal colon of F2 re-WT and re-PepT1$^{-/-}$ mice. **e,** Body weights of F3 re-WT and re-PepT1$^{-/-}$ mice during the DSS-treatment period. **f,** Colon weight/colon length ratio of F3 re-WT and re-PepT1$^{-/-}$ mice **g,** Spleen weight at F3. **h,** MPO activity in the distal colon of F3 re-WT and re-PepT1$^{-/-}$ mice. **i,** Body weights of F4 re-WT and re-PepT1$^{-/-}$ mice during the DSS-treatment period. **j,** Colon weight/colon length ratio of F4 re-WT and re-PepT1$^{-/-}$ mice. **k,** Spleen weight at F4. **l,** MPO activity in the distal colon of F4 re-WT and re-PepT1$^{-/-}$ mice. Data are presented as the mean ± SEM. Significance was determined by one-way ANOVA followed by a Bonferroni post-hoc test (n = 4–11 mice/group, *P<0.05; **P < 0.01; ***P< 0.001).

Supplementary Figure 7: Microbiota composition evolves with generation

a, c, e g, Bacterial-taxon–based analysis of fecal microbiota of F1 **(a)**, F2 **(c)**, F3 **(e)** and F4 **(g)** generations at the phylum level. **b, d, f, h,** LEfSe (LDA Effect Size) was used to investigate bacterial members that drive the differences in the fecal microbiota of re-WT and re-PepT1$^{-/-}$ mice at generation F1 **(b)**, F2 **(d)**, F3 **(f)**, F4 **(h)**. Left panel: Taxonomic cladogram obtained from LEfSe analysis of 16S rRNA sequences. Red, WT taxa; green, PepT1$^{-/-}$ taxa. The brightness of each dot is proportional to the size of its effect size. Right panel: LDA scores for the differentially altered taxa. Red, WT taxa; green, PepT1$^{-/-}$ taxa. Only taxa meeting an LDA significance threshold > 2.0 are represented.

Supplementary Figure 8: Correlation between inflammation parameters and OTUs. Analysis of correlations between OTUs and the inflammation parameters, colon weight/colon length ratio **(a)**, spleen weight **(b)**, percent of initial body weight **(c)** and colonic MPO activity **(d)**, in DSS-induced colitis mice across generations. Correlations for which R^2 = < -0.4 or >0.4 are shown.

Supplementary Data 1: OTU table summarized at the family level at generation F1. Relative abundance of bacterial families in F1 re-WT and re-PepT1$^{-/-}$ microbiota.

Supplementary Data 2: OTU table summarized at the family level at generation F2. Relative abundance of bacterial families in F2 re-WT and re-PepT1$^{-/-}$ microbiota.

Supplementary Data 3: OTU table summarized at the family level at generation F3. Relative abundance of bacterial families in F3 re-WT and re-PepT1$^{-/-}$ microbiota.

Supplementary Data 4: OTU table summarized at the family level at generation F4. Relative abundance of bacterial families in F4 re-WT and re-PepT1$^{-/-}$ microbiota.

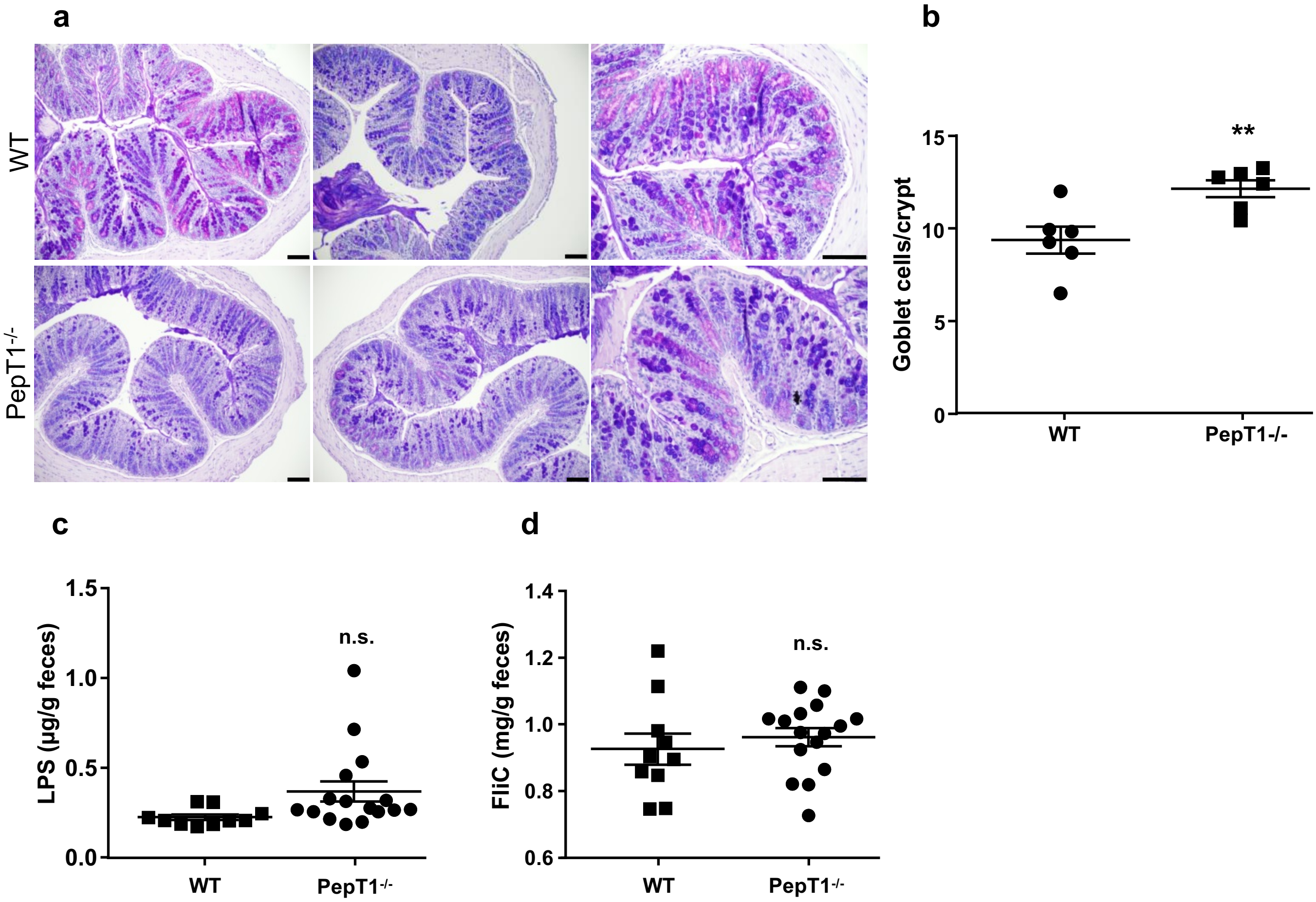

Supplementary Figure 1

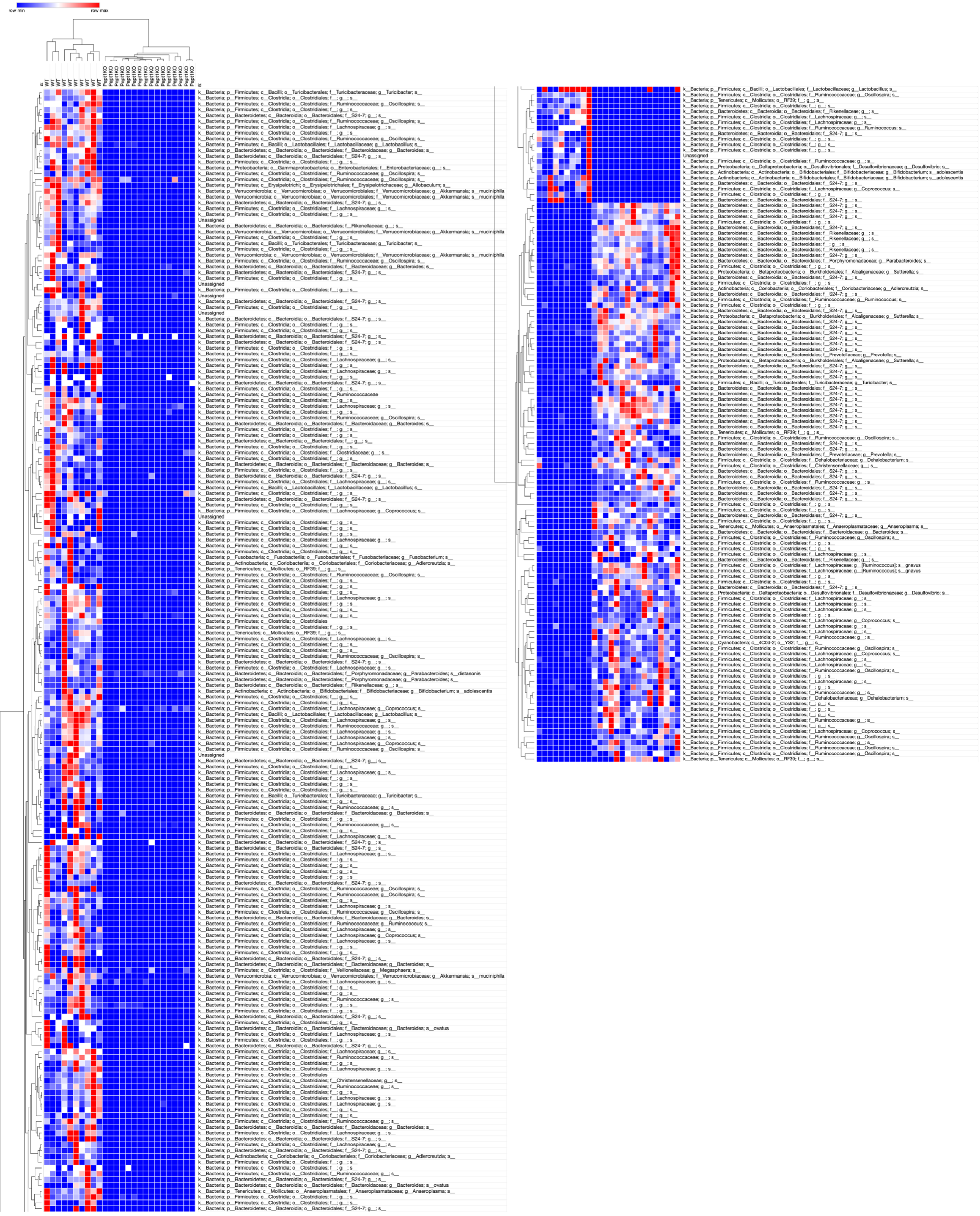

Supplementary Figure 2

a

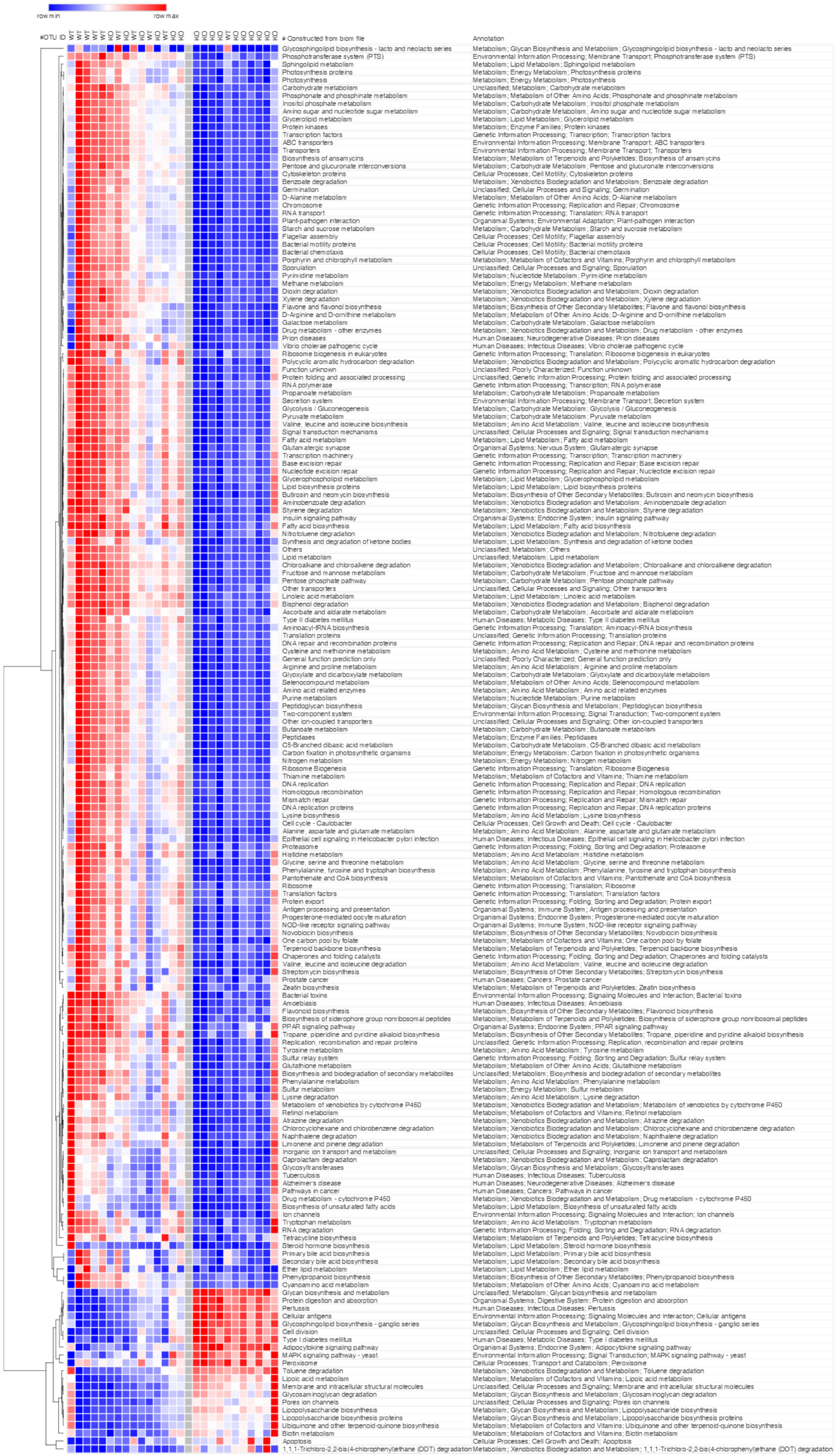

Supplementary figure 3

b

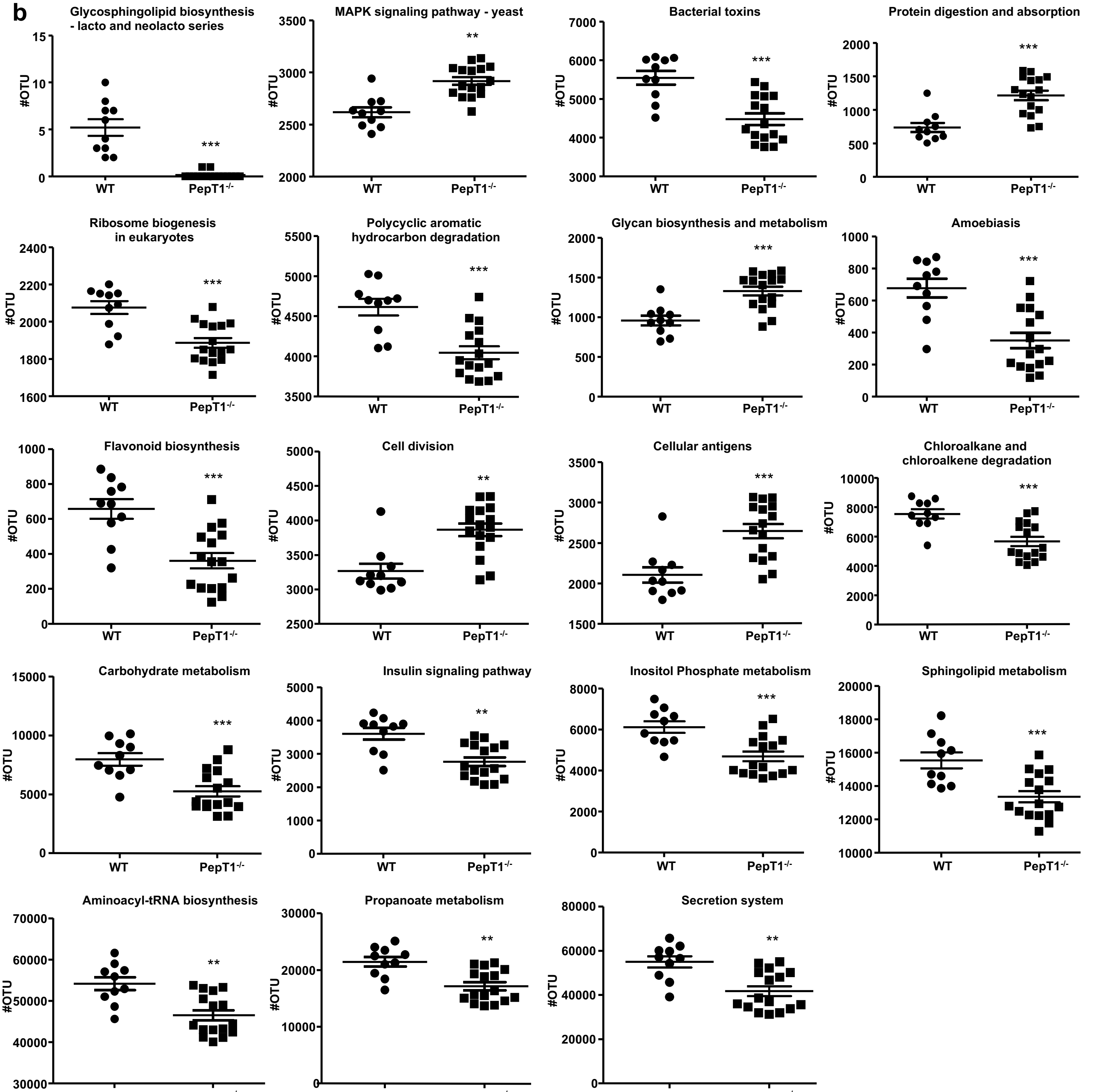

C

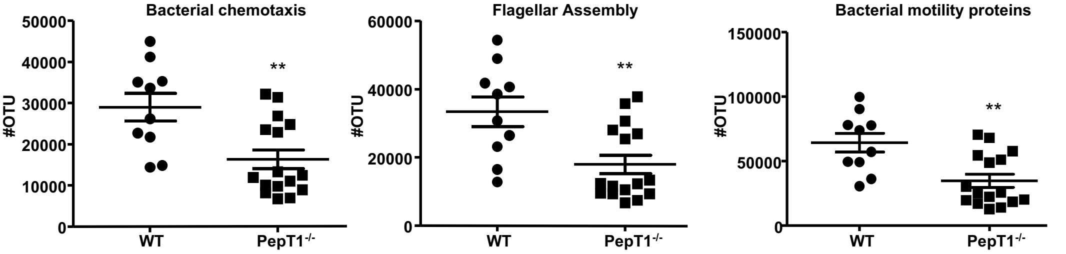

Supplementary figure 3

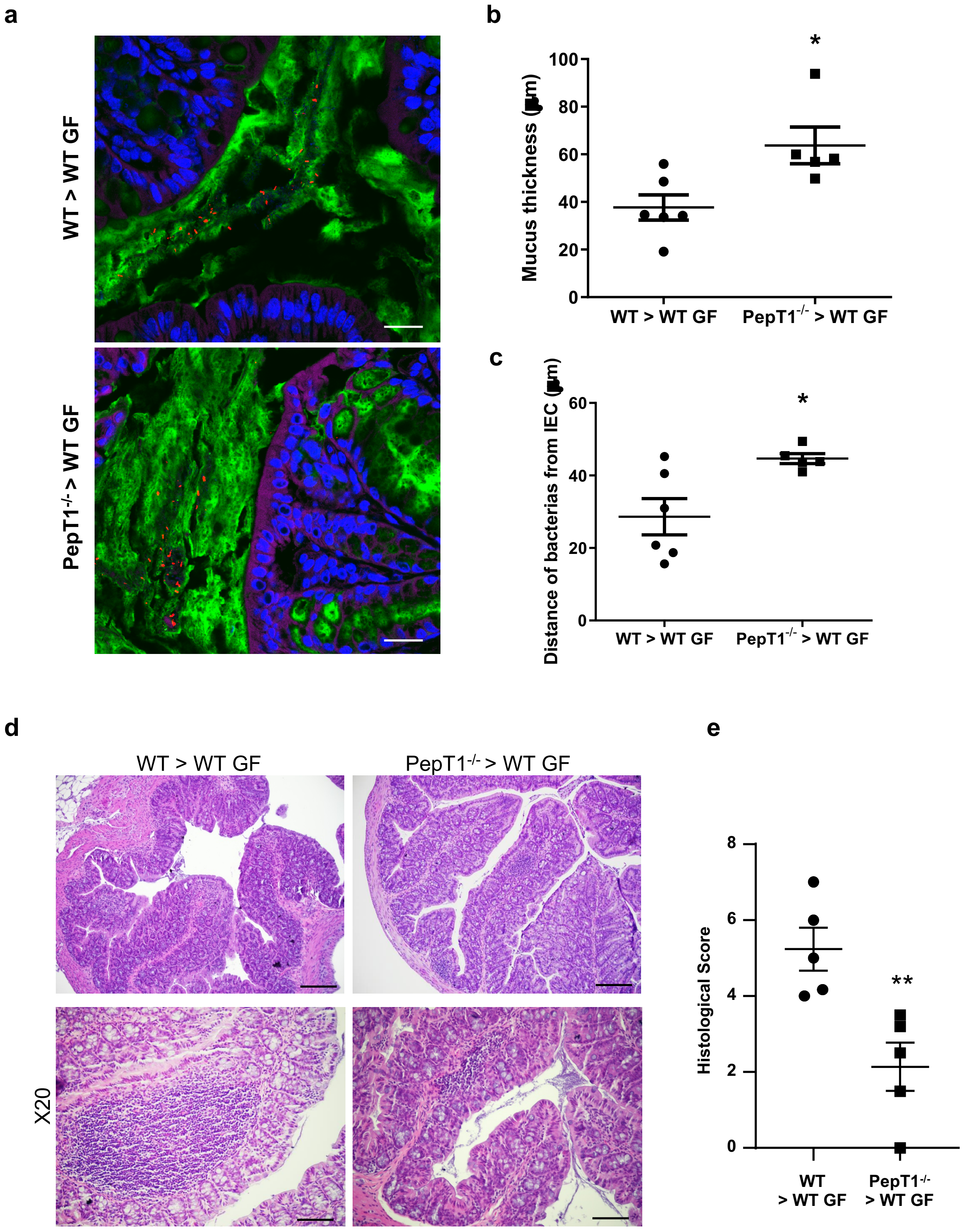

Supplementary figure 4

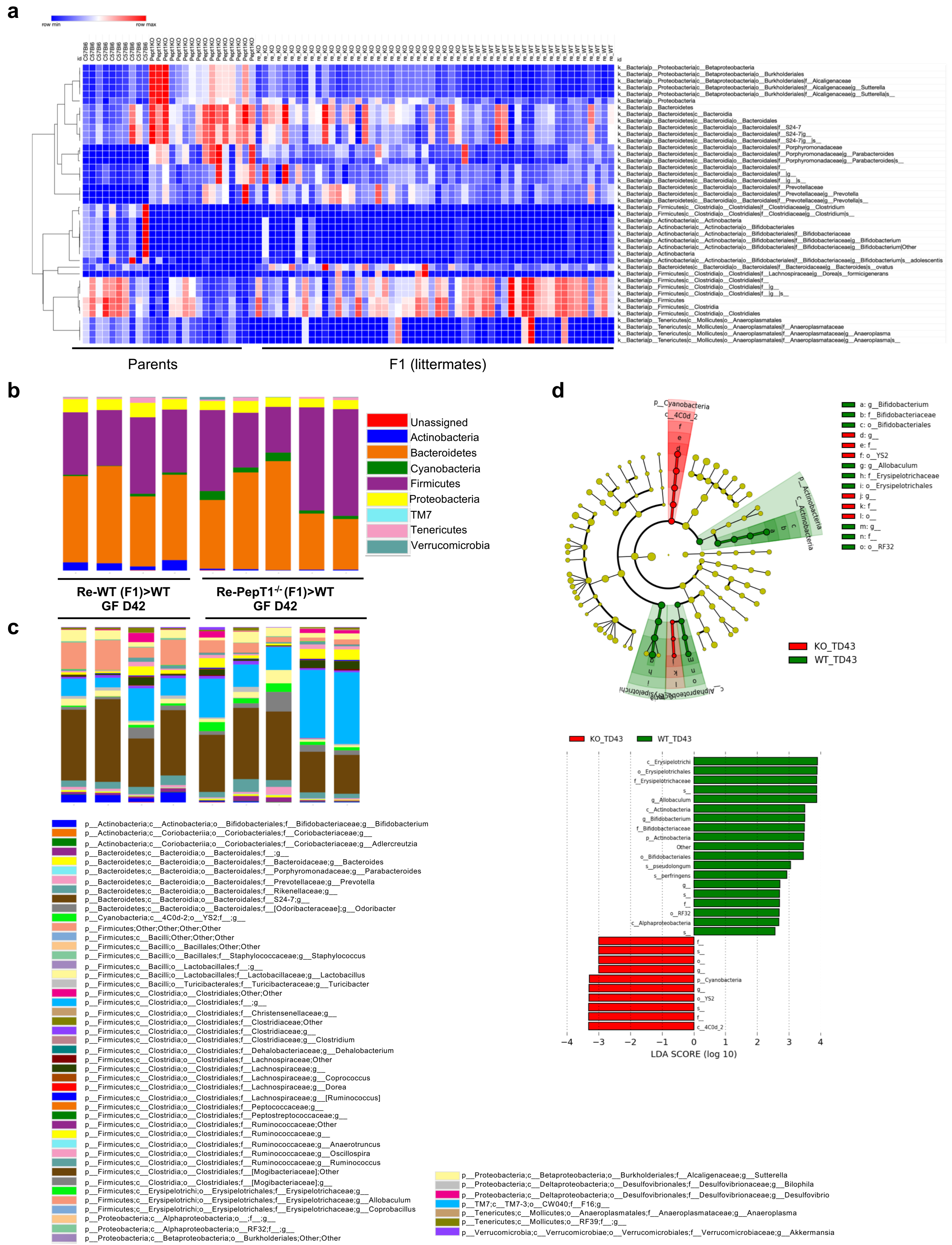

Supplementary figure 5

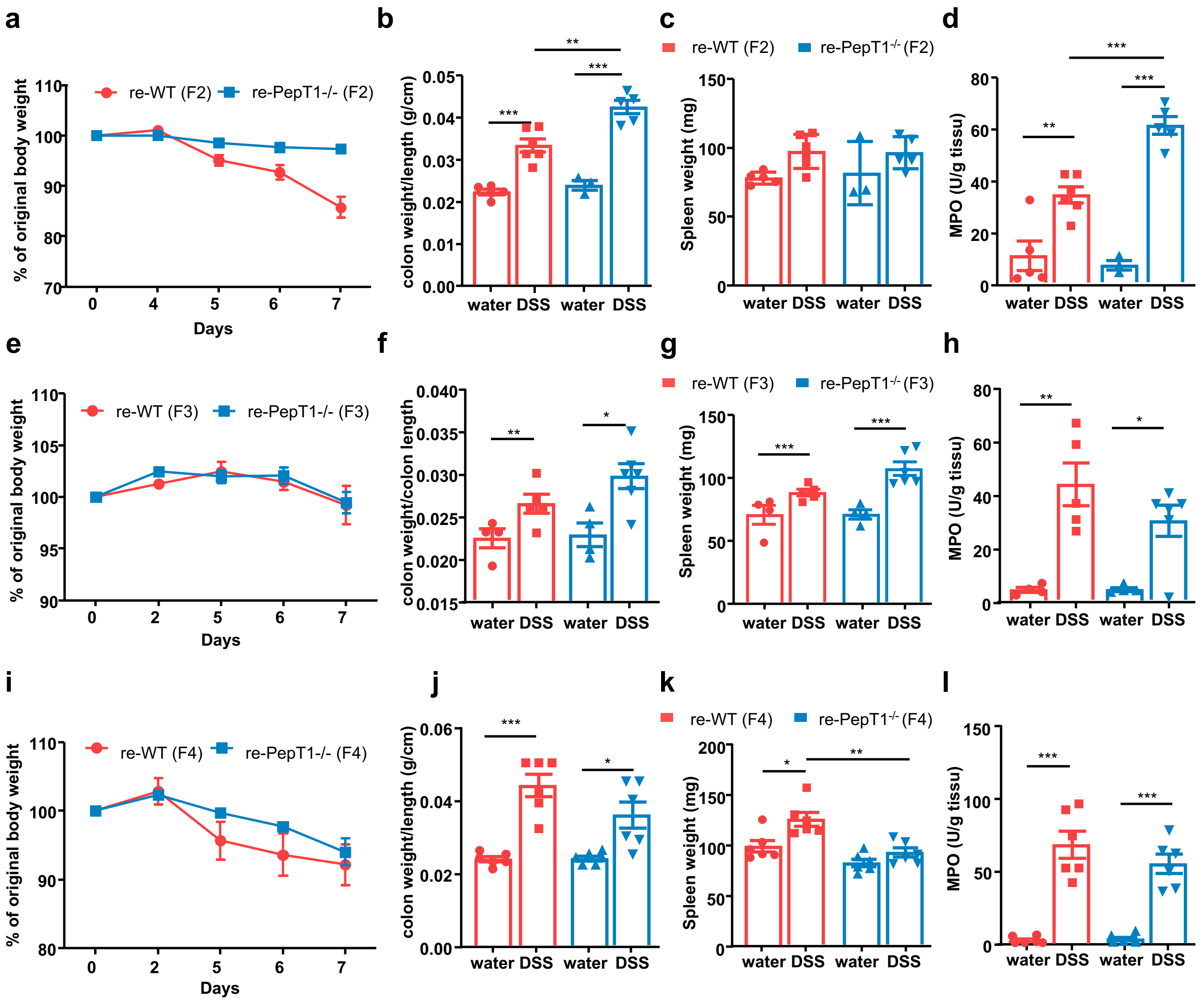

Supplementary figure 6

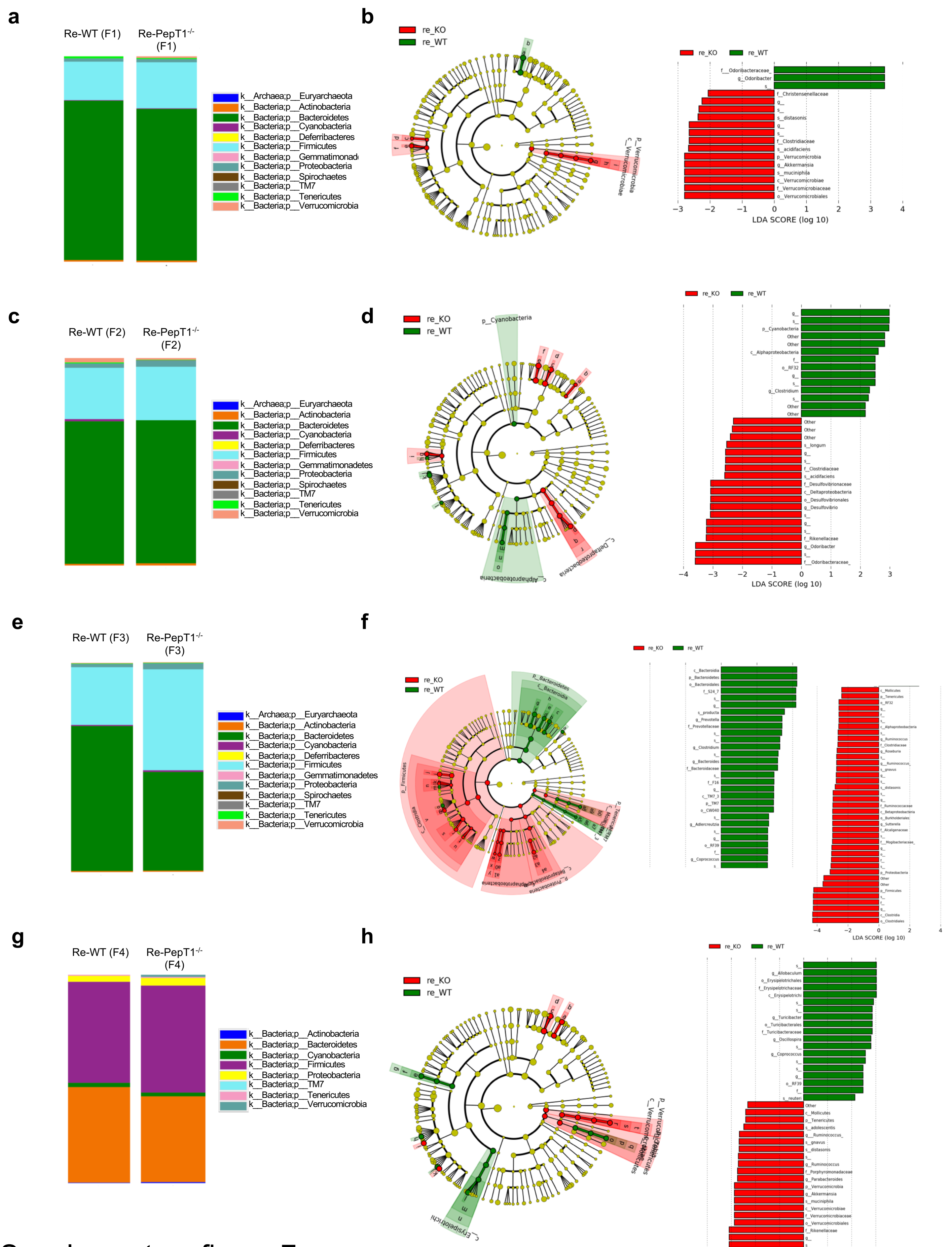

Supplementary figure 7

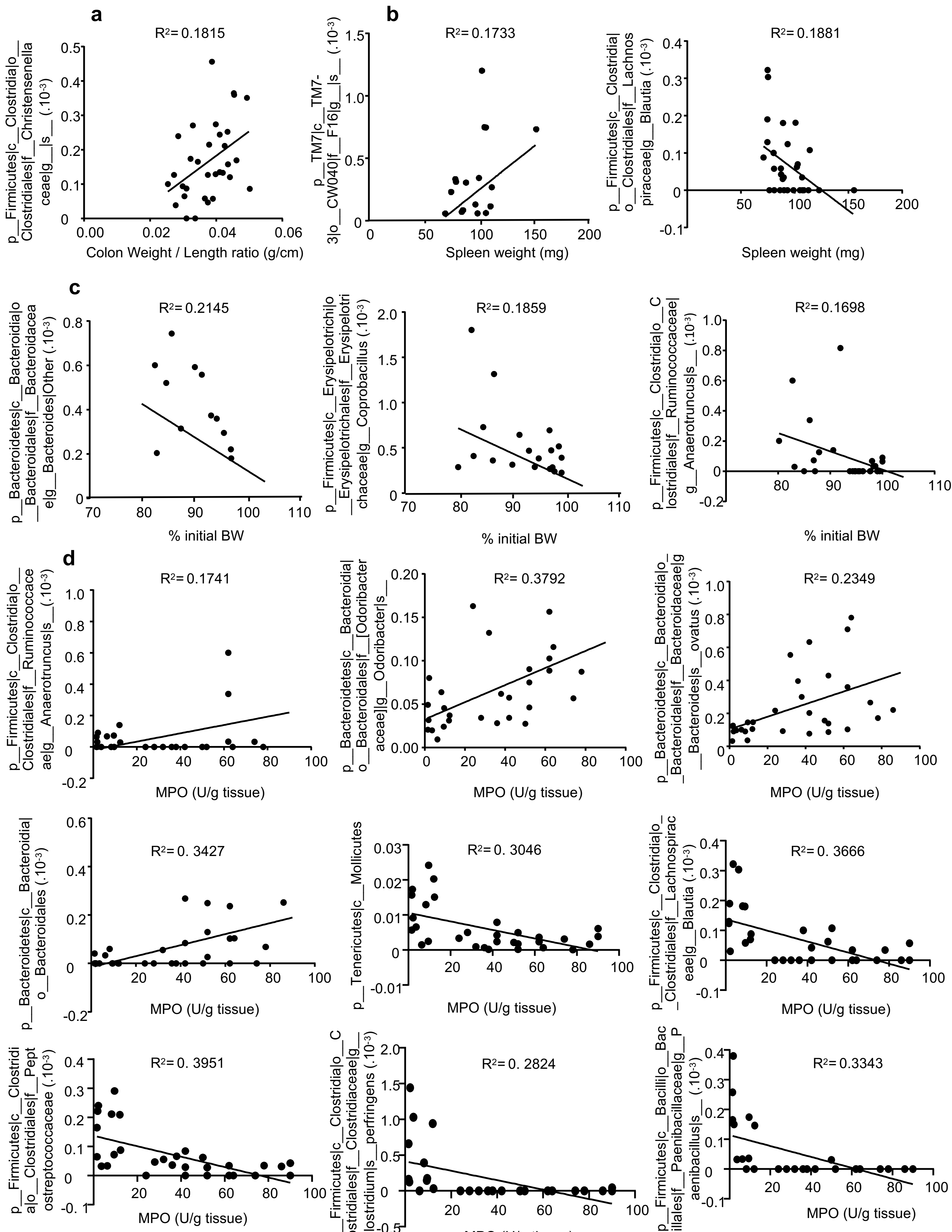

Supplementary figure 8
